# Supplementary material for: Spatial exclusion leads to “tug-of-war” ecological dynamics between competing species within microchannels
Source: PLoS Comput Biol. 2023 Dec 1;19(12):e1010868. doi: 10.1371/journal.pcbi.1010868 (PMC10718426; doi:10.1371/journal.pcbi.1010868)
Supplement: S1 Appendix — Section A. Calculation of the discrete mean first-passage time to fixation. We review the calculation of the probability and exact mean first-passage time for the discrete models discussed in this main text. Section B. The Fokker-Planck approximation. The Fokker-Planck approximations corresponding to the continuous limit of the exact discrete dynamics are derived. Section C. The Moran model. Details of the classical Moran model with fitness differences are summarized. The differences between results of the Fokker-Planck equation and the master equation are explored. Section D. The spatial exclusion model. Further details of the spatial exclusion model are clarified, including the MFPT of fixation conditioned on the success of one of the two species. Intermediate fitness differences are plotted. A comparison of the Fokker-Planck and master equation results is shown. Section E. Average probabilities and MFPT. The MFPT and probability of fixation are averaged over all initial fractional abundances for the one-boundary problem. Section F. Invasion into a fixated channel. The mathematical details of the inva.sion dynamics discussed above are outlined. Additional results on a minimal invasion from the side of the channel are also shown. Section G. The heuristic asymptotic MFPT approximation. The asymptotic in N behaviour of the maximal MFPT at the equiprobable takeover abundance is explored. A mathematical derivation involving an approximation to the MFPT of fixation is investigated. (PDF) [file pcbi.1010868.s001.pdf]

Appendix for 'Spatial exclusion leads to tug-of-war ecological dynamics between competing species within microchannels'

Jeremy Rothschild[1], Tianyi Ma[1,2], Joshua Milstein[1,2,*], and Anton Zilman[1,3,*]

1 Department of Physics, University of Toronto, Ontario, Canada
2 Department of Chemical and Physical Sciences, University of Toronto Mississauga, Ontario, Canada
3 Institute for Biomedical Engineering, University of Toronto, Ontario, Canada

* zilmana@physics.utoronto.ca, josh.milstein@utoronto.ca

Contents

A Calculation of the discrete mean first-passage time to fixation 1

B The Fokker-Planck approximation 3

C The Moran model 5
C.1 continued . . . 5
C.2 Neutral . . . 5
C.3 Fitness differences . . . 6

D The spatial model 7
D.1 Neutral . . . 8
D.2 Fitness differences . . . 10
D.3 Comparing the master equation to Fokker-Planck . . . 11
D.4 MFPT conditioned on the success of one species . . . 11

E Average probabilities and MFPT 13

F Invasion into a fixated channel 14
F.1 Invasions from the edge of the channel . . . 17

G The heuristic asymptotic MFPT approximation 19

A Calculation of the discrete mean first-passage time to fixation

As outlined in the main text, the populations that we study are represented by their abundances, i.e. the number of individuals present in the system for each species. At time t, the state of the system with multiple species is determined by the abundance n for each species. Birth and death dynamics under the influence of demographic noise govern the dynamics of these abundances, that is to say that random birth and death events cause stochastic fluctuations of the abundances in the populations. Given this stochastic description of the population, each species population abundance is a random variable which combine in a joint probability distribution, denoted $p(\{n\}; t)$.

The system we study has a number of constraints that characterize the organization of the population abundances. Namely, the ecosystem has a fixed total population size,

N, which is split between two species whose abundances are n and $N-n$ respectively. Consequently, the state of this two-species system is defined by the abundance of either species since one abundance determines the other, and the increase of one species, $n \to n+1$, coincides with the decrease of the other, $N-n \to N-n-1$. In this case, the probability distribution $p(n;t)$ is one-dimensional.

The population abundance of a species can change by increasing (birth) or decreasing (decrease) the number of individuals, with the probability of birth or death in the population n in an interval of time Δt denoted as $T^{+}(n \to n+1, \Delta t)$ or $T^{-}(n \to n-1, \Delta t)$, respectively [1,2]. The evolution of the probability $p(n;t)$ is governed by a one-dimensional forward master equation (ME)

$$\begin{aligned} p(n;t+\Delta t) = T^{-}(n+1 \to n, \Delta t)p(n+1;t) + T^{+}(n-1 \to n, \Delta t)p(n-1;t) \\ + \left(1 - T^{+}(n \to n+1, \Delta t) - T^{-}(n \to n-1, \Delta t)\right) p(n;t). \quad (1) \end{aligned}$$

The continuous time master equation that describes the evolution of the probability distribution of n is the differential form for Eq 1

$$\partial_t p(n;t) = r_N^{+}(n-1)p(n-1;t) + r_N^{-}(n+1)p(n+1;t) - \left(r_N^{+}(n) + r_N^{-}(n)\right) p(n;t), \quad (2)$$

where $\lim_{\Delta t \to 0} T^{\pm}(n \to n+1, \Delta t)/\Delta t = r_N^{\pm}$ are the rates of birth (+) and death (−) [3].

This set of differential equations can be written in matrix form

$$\dot{\mathbf{P}} = \mathbf{M}\mathbf{P}(t) \quad (3)$$

where $\mathbf{M}$ is a tridiagonal matrix with the off-diagonal elements $(\mathbf{M})_{n,n\mp 1} = r_N^{\pm}(n \mp 1)$, and the diagonal elements $(\mathbf{M})_{n,n} = -\left(r_N^{+}(n) + r_N^{-}(n)\right)$. In this matrix notation, the vector of probabilities is $\mathbf{P}^T(t) = (p(0;t), p(1;t), ..., p(N;t))$. The solution to Eq 3 with an initial probability distribution $\mathbf{P}(0)$ is, in matrix form,

$$\mathbf{P}(t) = e^{\mathbf{M}t}\mathbf{P}(0). \quad (4)$$

At steady state, the left-hand side (LHS) of Eq 3 is zero; given that the system is finite, the matrix $\mathbf{M}$ has any zero eigenvalues and the system has at least one steady-state distribution, $p_{ss}(n)$. If any of these steady-state distributions is a Kronecker delta function $p_{ss}(n) = \delta_{n,f}$, then the state f is an absorbing state of the system, and no probability leaves this state once it has entered [4]. These absorbing states can be found directly from the evaluation of the matrix: If the only term in a column of $\mathbf{M}$ is the diagonal, then the state corresponding to the column is an absorbing state. In the case of two populations competing for dominance in a fixed total population size N, the only absorbing states are $n = 0$ and $n = N$.

For the calculation of the mean first-passage time (MFPT), it is convenient to define a transition matrix excluding all absorbing states is $\tilde{\mathbf{M}}$ where all rows and columns corresponding to the absorbing states, $F = \{f\}$, have been removed. Similarly, removing all the absorbing states from the probability distribution returns a vector of the unnormalized probability of the non-absorbing states, $\tilde{\mathbf{P}}(t)$. The new transition matrix and unnormalized probability vector excluding the absorbing states can replace $\mathbf{P}$ and $\mathbf{M}$ in Eq 4. This new matrix equation describes the dynamics of the system losing probability to the absorbing states.

In the presence of many absorbing states $F = \{f_1, f_2, ...\}$, we are interested in the absorption/splitting probabilities, denoted as the vector $\mathbf{P}_F = (p_{ss}(f_1), p_{ss}(f_2), ...)$, starting from an initial distribution $\tilde{\mathbf{P}}(0)$. Given that the probability of finishing in an absorbing state is equal to the integral in time of the probability flux into that state [5], the probability distribution vector for finishing in the absorbing states is calculated as

$$\mathbf{P}_F = \int_0^{\infty} dt \mathbf{A} e^{\tilde{\mathbf{M}}t}\tilde{\mathbf{P}}(0). \quad (5)$$

$\mathbf{A}$ is a matrix of dimension $(|F|, N-|F|)$ whose rows correspond to the removed rows of $\tilde{\mathbf{M}}$, that is, vectors of transition rates in the states F. Only the exponential in Eq 5 depends on time, which simplifies the equation to

$$\begin{aligned}\mathbf{P}_F &= \mathbf{A}(\int_0^\infty dt e^{\tilde{\mathbf{M}}t})\tilde{\mathbf{P}}(0) \\ &= \mathbf{A}\tilde{\mathbf{M}}^{-1}(\mathbb{I} - e^{\tilde{\mathbf{M}}\infty})\tilde{\mathbf{P}}(0) \\ &= \mathbf{A}\tilde{\mathbf{M}}^{-1}\tilde{\mathbf{P}}(0). \end{aligned} \tag{6}$$

The probability density of the first passage times into each absorbing state f_i is then [5]

$$\mathcal{P}_i(t) = \frac{1}{(\mathbf{P}_F)_i}\left(\mathbf{A}e^{\tilde{\mathbf{M}}t}\tilde{\mathbf{P}}(0)\right)_i. \tag{7}$$

The MFPT starting from an initial distribution $\tilde{\mathbf{P}}(0)$ is

$$\begin{aligned}\tau_{f_i} &= \int_0^\infty t\mathcal{P}_i(t)dt \\ &= \frac{1}{(\mathbf{P}_F)_i}\left(\mathbf{A}(\int_0^\infty dt e^{\tilde{\mathbf{M}}t}t)\tilde{\mathbf{P}}(0)\right)_i \\ &= \frac{1}{(\mathbf{P}_F)_i}\left(\mathbf{A}(\tilde{\mathbf{M}}^{-1})^2\tilde{\mathbf{P}}(0)\right). \end{aligned} \tag{8}$$

To calculate the time starting from an initial state $\tau_{f_i}(n_0)$, the probability is initialized as a Kronecker delta $\tilde{\mathbf{P}}(0) = \delta_{i,n_0}$.

B The Fokker-Planck approximation

Analogous to the forward master equation, we can describe the evolution of $p(n,t|n_0,t_0)$ (the probability to be at state n at time t having started at the state n_0 at time t_0) by the backward master equation

$$\begin{aligned}p(n,t|n_0,t_0) &= T^+(n_0,\Delta t)p(n,t|n_0+1,t_0+\Delta t) + T^-(n_0,\Delta t)p(n,t|n_0-1,t_0+\Delta t) \\ &\quad - \left(1 - T^+(n_0,\Delta t) - T^+(n_0,\Delta t)\right)p(n,t|n_0,t_0+\Delta t), \\ p(n,t|n_0,t_0) &= T^+(n_0,\Delta t)p(n,t-\Delta t|n_0+1,t_0) + T^-(n_0,\Delta t)p(n,t-\Delta t|n_0-1,t_0) \\ &\quad - \left(1 - T^+(n_0,\Delta t) - T^+(n_0,\Delta t)\right)p(n,t-\Delta t|n_0,t_0). \end{aligned} \tag{9}$$

Here, the stationarity of the transition probabilitiew - their lack of a dependence on the absolute time - justifies the transformation $p(n,t|n_0,t_0+\Delta t) = p(n,t-\Delta t|n_0,t_0)$. In the continuous time approximation (limit of small Δt), the differential form of the backward master equation is

$$\partial_t p_n(n_0;t) = r_N^+(n_0)\left(p_n(n_0+1;t) - p_n(n_0;t)\right) + r_N^-(n_0)\left(p_n(n_0-1;t) - p_n(n_0;t)\right) \tag{10}$$

where we have rewritten $p(n,t|n_0,0) = p_n(n_0;t)$. Recall that $r_N^+(n)$ and $r_N^-(n)$ are the rate at which the species abundance n increase and decrease by 1 respectively.

So far, we've used the discrete species abundance n as the variable which defines the state of the system. However, it is often convenient to approximate the master equation in the continuum limit if a large characteristic system size, such as the total population size, exists.

We assume that the system size is large, $N \gg 1$, and define y to be the corresponding continuous limit of n which can be rescaled by the system size, with $f = y/N$ as the fractional abundance. In this continuous formulation, the absorbing states of the two population competition are now at $f = 0$ and $f = 1$. The discrete probabilities are transformed into probability densities of the continuous variable $Np_n(n_0;t) \to P_f(f_0;t)$, with similar transformations of the re-scaled rates, $r_N^{+/-}(n) \to r^{+/-}(f)$ where $f \in [0,1]$.

Written in the continuous variables, the backwards master equation, Eq 10, becomes

$$\partial_t P_f(f_0;t) = r^+(f_0)\left(P_f(f_0+1/N;t) - P_f(f_0;t)\right) + r^-(f_0)\left(P_f(f_0-1/N;t) - P_f(f_0;t)\right). \quad (11)$$

A Taylor expansion in orders of $1/N$ of the terms in this equation gives $P_f(f_0 \pm 1/N;t) = P_f(f_0;t) \pm \frac{1}{N}\partial_{f_0}P_f(f_0) + \frac{1}{2N^2}\partial_{f_0}^2 P_f(f_0) + \mathcal{O}(N^{-3})$. Using this expansion at steady state $\partial_t P_f(f_0;t) = 0$, the differential form of Eq 11 is the steady-state Fokker-Planck (FP) equation

$$0 = \frac{A(f_0)}{N}\partial_{f_0}P_f(f_0) + \frac{B(f_0)}{2N^2}\partial_{f_0}^2 P_f(f_0) + \mathcal{O}(1/N^3), \quad (12)$$

where

$$A(f_0) = r^+(f_0) - r^-(f_0) \quad (13)$$

$$B(f_0) = r^+(f_0) + r^-(f_0) \quad (14)$$

are respectively the drift and diffusion terms. The probability to fixate at f from an initial position f_0, $P_f(f_0)$, can be calculated from Eq 12 by solving the differential equation with an appropriate choice of boundary condition. For instance, the correct boundary conditions to find the probability that the fractional abundance fixates at $f = 1$ in our two population competition are $P_1(1) = 1$ and $P_1(0) = 0$.

In conjuncture with calculating the probability of fixation, the times in which a random process first reaches a certain threshold of abundance can be calculated in this continuous formulation. Given a set of absorbing states which correspond to the fixation of populations, the mean-first passage time of absorption into any of the absorbing states $\tau(n_0)$ marks the average timescale in which a population out-competes another starting from an initial abundance of n_0. This first-passage time is described by the evolution equation [2]

$$\begin{aligned}\tau(n_0) = \Delta t + T^+(n_0 \to n_0+1, \Delta t)\tau(n_0+1) + T^-(n_0 \to n_0-1, \Delta t)\tau(n_0-1) \\ + \left(1 - T^+(n_0 \to n_0+1, \Delta t) - T^-(n_0 \to n_0-1, \Delta t)\right)\tau(n_0)\end{aligned} \quad (15)$$

In the continuum limit, the equation for the mean first-passage time is

$$-1 = r^+(f_0)\left(\tau(f_0+1/N) - \tau(f_0)\right) + r^-(f_0)\left(\tau(f_0-1/N) - \tau(f_0)\right). \quad (16)$$

A Taylor expansion of the $\tau(f_0 \pm 1/N) = \tau(f_0) \pm \frac{1}{N}\tau'(f_0) + \frac{1}{2N^2}\tau''(f_0) + \mathcal{O}(N^{-3})$ transforms Eq 16 into

$$-1 = \frac{A(f_0)}{N}\tau'(f_0) + \frac{B(f_0)}{2N^2}\tau''(f_0). \quad (17)$$

As mentioned above, the two population system entertains two absorbing states at $f = 0$ and $f = 1$. The mean-time to fixate at either of these absorbing states from an initial position f_0, $\tau(f_0)$, can be calculated from Eq 12 by solving the differential equation with the appropriate choice of boundary condition, $\tau(0) = \tau(1) = 0$. Consequently, the integral form of the solution to the Fokker-Planck Eq 17 is [2]

$$\tau(f) = 2N^2 \frac{\left(\int_0^x dy e^{U(y)}\right)\int_f^1 dy' e^{U(y')}\int_0^{y'} dz \frac{e^{-U(z)}}{B(z)} - \left(\int_f^1 dy e^{U(y)}\right)\int_0^x dy' e^{U(y')}\int_0^{y'} dz \frac{e^{-U(z)}}{B(z)}}{\int_0^1 dy e^{U(y)}} \quad (18)$$

where

$$U(f) = -2N \int_0^x dx' \frac{A(x')}{B(x')} \tag{19}$$

is often referred to as the Fokker-Planck effective potential.

As well as studying the MFPT to fixation through any absorbing state, the splitting MFPT to reach an individual absorbing state can be calculated. This directional MFPT, $\tau_f(f_0)$, describes the timescale of abundance fluctuations in a particular direction: from an initial abundance to one of the two absorbing boundaries. The differential equation that calculates the directional MFPT through absorbing state f is

$$-P_f(f_0) = \frac{A(f_0)}{N}(P_f(f_0)\tau_f(f_0))' + \frac{B(f_0)}{2N^2}(P_f(f_0)\tau_i(f_0))''. \tag{20}$$

This equation depends on the probability of fixating at the specific absorbing state, $P_f(f_0)$, which is a solution of Eq 12. Note that we have replaced the notation of the initial abundance f_0 to f in the rest of this work.

C The Moran model

C.1 continued

The paradigmatic Moran model describes the natural selection and abundance drift of strains in a fixed population as a stochastic process. The dynamics between the two species can be either neutral or entertain fitness differences.

C.2 Neutral

An assumption of inter-specific neutrality supposes that species are functionally equivalent, i.e. the probability that an individual is selected for birth (or death) is equivalent for all species. In the truly neutral case without fitness differences between the species, the probability for the species with n individuals to increase in size is the product of the probability that a member of that species is selected for a birth event (n/N) and the probability that the other species is selected for a death event $((N-n)/N)$. Consequently, the probabilities of abundance transitions in a discrete timestep Δt in the Moran model are

$$\begin{aligned} T^+(n \to n+1) &= \frac{n}{N}\frac{N-n}{N} \\ T^-(n \to n-1) &= \frac{N-n}{N}\frac{n}{N}. \end{aligned} \tag{21}$$

which are characteristically identical in the Moran model. The rates of these transitions are

$$\begin{aligned} r_N^+(n \to n+1) &= rn\left(1 - \frac{n}{N}\right) \\ r_N^-(n \to n-1) &= rn\left(1 - \frac{n}{N}\right), \end{aligned} \tag{22}$$

where the basal growth rate has been rescaled by $r = 1/(N\Delta t)$. The rescaling of r results in continuous rates that resemble the classical logistic equation for populations of $n = Nf$ individuals. The corresponding continuous rates in the continuous framework are

$$\begin{aligned} r^+(f) &= rNf(1-f) \\ r^-(f) &= rNf(1-f). \end{aligned} \tag{23}$$

The rates of the discrete transitions can be used in Eq 6-8 to solve for the probability and MFPT numerically, however analytical approximations for these quantities are also obtained by solving Eq 12-20. The drift and diffusion terms (Eq 13-14) of the FP expression are

$$\begin{aligned} A(f) &= 0 \\ B(f) &= 2rNf(1-f). \end{aligned} \tag{24}$$

Exceptionally, the drift term is zero everywhere in the Moran model; this simplifies the calculation of the probability as well as that of the MFPT. Solving the differential Eq 12 for the probability of fixation at 1 of a species starting at f fractional abundance gives

$$P_1(f) = f. \tag{25}$$

This is a classical result: the probability for a species to successfully takeover the system in the Moran model is equal to its fractional abundance.

As for the MFPT to fixation, a linear scaling with system size, N, emerges from the solution of Eq 17

$$-1 = \frac{rf(1-f)}{N}\tau''(f) \quad \rightarrow \quad \tau(f) = -N\frac{1}{r}\left[(1-f)\log(1-f) + f\log(f)\right] \tag{26}$$

The MFPT of the Moran model in other derivations is shown to scale with the square of the system size, N^2 [6, 7]. This statement is consistent with the result of Eq 26 given that we have rescaled the basal growth rate by $r = 1/(N\Delta t)$.

From Eq 20, the directional MFPT for a species with f fractional abundance to take over the population is

$$-f = \frac{rf(1-f)}{N}(f\tau_1(f))'' \quad \rightarrow \quad \tau_1(f) = -N\frac{(1-f)\log(1-f))}{r(1+f)}. \tag{27}$$

Like the MFPT of fixation, the directional MFPT depends linearly on the total population.

C.3 Fitness differences

A Moran model with relative fitness differences assumes that one of the two species has a selective advantage. Relative fitness differences/selective advantages between the species can be incorporated into the model by appropriately changing the probability of selecting a species for a birth/death. In the simplest form of this selective model, the selective advantage is only present in the birth event of a species. By assuming that a fitness difference w - or coefficient of selection in evolutionary dynamicw - exists between the species, the rates of the Moran model with selection are

$$\begin{aligned} r_N^{\text{Moran},+}(n) &= rw\frac{n(N-n)}{N+(w-1)n} \\ r_N^{\text{Moran},-}(n) &= r\frac{n(N-n)}{N+(w-1)n}, \end{aligned} \tag{28}$$

where $s > 1$ indicates a fitness advantage. Unlike the neutral Moran model, these transition probabilities are not identical given the asymmetry conferred by the selective advantage: an individual from one of the species is more likely to give birth than an individual of the other species. The rates corresponding to these transitions are

$$r_N^+(n \rightarrow n+1) = rw\frac{n(N-n)}{wn+(N-n)} \tag{29}$$

$$r_N^-(n \rightarrow n-1) = r\frac{n(N-n)}{wn+(N-n)}, \tag{30}$$

and the rates in the continuous framework are

$$r^{+}(f) = rNw\frac{f(1-f)}{1+(w-1)f} \tag{31}$$

$$r^{-}(f) = rN\frac{f(1-f)}{1+(w-1)f}. \tag{32}$$

As with the neutral model, the rates of the discrete transitions can be replaced in Eq 6-8 to solve this equation numerically. An analytical approximation is obtained Eq 12 for the probability of fixation. The drift and diffusion terms (Eq 13-14) of the FP expression are

$$A(f) = rN(w-1)\frac{f(1-f)}{1+(w-1)f} \tag{33}$$

$$B(f) = rN(w+1)\frac{f(1-f)}{1+(w-1)f}, \tag{34}$$

which leads to a probability of fixation of

$$P_1(f_0) = \frac{1-e^{-2Nf_0\frac{w-1}{w+1}}}{1-e^{-2N\frac{w-1}{w+1}}}. \tag{35}$$

Interestingly, the discrete master equation with a fitness difference can also be solved analytically, resulting in a probability of fixation

$$P_{n=N}(n_0) = \frac{1-w^{-n_0}}{1-w^{-N}} \tag{36}$$

from an initialized state n_0 [7]. In Fig 1A, the discrete solution is shown to approximately equal the Fokker-Planck approximation when $w \approx 1$. This is where both Eqs 35 and 36 are closest, i.e. $e^{2\frac{w-1}{w+1}} \approx w$. The discrepancy between the probability of fixation of the master equation approach and the Fokker-Planck approximation remains small at large N, see Figs 1B and 1D.

Unfortunately, neither of the solutions to the Fokker-Planck equation for the MFPT of fixation (Eq 17) or the directional MFPT (Eq 20) have simple algebraic expressions. However, it is advantageous to solve these differential equations numerically for large population sizes N compared to solving Eq 8 numerically given that the computation and memory usage of finding large matrix inverses is expensive. We show below in Section D.3 that the solutions to the master equation and Fokker-Planck approximations follow each other closely, in particular at large total population N.

D The spatial model

As described in the main text, the spatial model is inspired by the Moran model described above, however it aims to describe the competition of bacteria aligned in a channel. Similar to the Moran model, we consider a system of N individuals of two species; a species increases by one individual in any time step by having an individual randomly selected to duplicate (birth event) whilst simultaneously removing an individual of the other species from the system (death event) in the same step.

However, the spatial model has an added organizational structure that differentiates it from the Moran model; the individuals are constrained to a one-dimensional channel with both ends open. Contrary to the Moran model, the transition rates of the populations now depend on the spatial locations of the cells. Each species is segregated to either

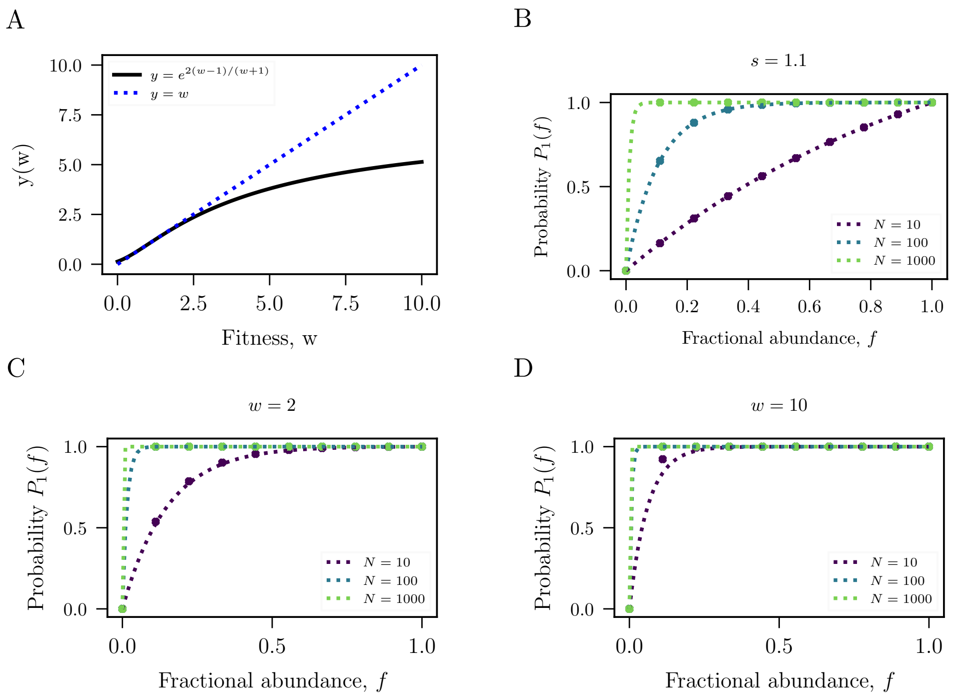

Fig 1. Comparison between probability of fixations calculated by the master equation and the Fokker-Planck solution for the Moran model with fitness differences w. **(A)** Plot of the terms which differentiate the solution of Fokker-Planck approximation in Eq 35 (blue dotted line) from the solution of the master equation (black line). The two terms $y(w)$ are in agreement around $w = 1$. (**B, C, D**): Comparison of the probabilities for different w and N for solutions of FP (dotted line) and ME (scatter points). **(B)** For $w = 1$ and **(C)** FP and ME solutions are in agreement. **(D)** At $w = 10$, differences are noticeable between ME and FP solutions at low N, although the shapes of the probability function remain the same.

side of the channel with n number of individuals belonging to the species on the left side of the lane. The species on the LHS of the channel grows in numbers when any individual of that species gives birth to the right, pushing individuals of the other species out of the channel. Likewise, their numbers decrease in channel when the species on the right-hand side (RHS) of the channel grow to the left. In this tug-of-war, the boundary at n individuals determines the state of the system, moving to the right (left) as n increase (decreases).

D.1 Neutral

Given no inter-species differences in terms of selection for growth/birth, the probability of any individual being selected to give birth is $1/N$ under the assumption that the basal birth rate is not location dependent. The probability of pushing out cells on either side to make room for progeny will depend on the location of the cell giving birth; a cell at position i will have a probability of $(i-1)/(N-1)$ of pushing to the right and a probability of $(N-i)/(N-1)$ of pushing to the left. The transition probabilities are

then

$$T^{+}(n) = \sum_{i=1}^{n} \frac{1}{N} \frac{i-1}{N-1} = \frac{n(n-1)}{2N(N-1)} \tag{37}$$

$$T^{-}(n) = \sum_{i=n+1}^{N} \frac{1}{N} \frac{N-i}{N-1} = \frac{(N-n)(N-n-1)}{2N(N-1)}, \tag{38}$$

where the probability of the boundary at n moving to the right (T^{+}) is equal to the sum of the probability that each individual of the species on the LHS gives birth to the right. Similarly, the probability of the boundary at n moving to the left (T^{-}) is equal to the sum of the probability that each individual of the species on the RHS gives birth to the left.

A more general version of the neutral model is evaluated in *Koldaeva et al.*, where the probabilities of the boundary moving at n are

$$T^{+}(n) = \frac{n(N-1-m(N-n)}{2N(N-1)} \tag{39}$$

$$T^{-}(n) = \frac{(N-n)(N-1-mn)}{2N(N-1)}, \tag{40}$$

where m is a parameter representing the magnitude of the biased growth towards either end of the channel. When $m = 0$, the growth of the progeny is unbiased. At $m = 1$, we recover our transition probabilities where there is strong bias of growth towards the ends of the channel.

The corresponding rates for the boundary moving to the right and left are

$$r_N^{+}(n) = r\frac{n(n-1)}{2N} \tag{41}$$

$$r_N^{-}(n) = r\frac{(N-n)(N-n-1)}{2N} \tag{42}$$

where the basal rate has been redefined as $r = 1/(N-1)\Delta t$. These rates in their continuous description, $f = n/N$, are

$$r^{+}(f) = rN\frac{f^2}{2} \tag{43}$$

$$r^{-}(f) = rN\frac{(1-f)(1-f)}{2}. \tag{44}$$

In the Fokker-Planck approximation, the drift and diffusion terms are respectively

$$A(f) = rN\frac{2f-1}{2} \tag{45}$$

$$B(f) = rN\frac{2f^2-2f+1}{2}. \tag{46}$$

A notable difference compared with the neutral Moran model (Eq 33) is that the drift term in the neutral spatial model is non-zero. This complicates the calculation of algebraic solutions for the probability and MFPT of fixations in Eqs 12 and 20. Nonetheless, the solution for the probability of fixation can be expressed in algebraic form,

$$P_1(f) = \frac{\operatorname{erf}\left(\sqrt{N}(2f-1)/2\right)}{2\operatorname{erf}\left(\sqrt{N}/2\right)} + \frac{1}{2} \tag{47}$$

where erf is the error function.

Because neither of the solutions to the Fokker-Planck equation for the MFPT of fixation (Eq 17) or the directional MFPT (Eq 20) have simple algebraic expressions, we instead solve them numerically.

D.2 Fitness differences

Like the Moran model, fitness differences change the probabilities of selection for a birth event. An individual of species 1 with fitness advantage w is selected for giving birth with probability $p_1^+ = w/[(N-n)+wn]$, whereas the probability for an individual of species 2 is $p_2^+ = 1/[(N-n)+wn]$. Following the birth, the progeny at location i makes room for itself by pushing the cells on either side of its progenitor outwards according to the same rules as the neutral model. The probabilities of the boundary at n moving to the right and left are

$$T^+(n) = \sum_{i=1}^{n} \left(\frac{w}{(N-n)+wn} \right) \frac{i-1}{N-1} = \frac{wn(n-1)}{2(N-1)[(N-n)+wn]} \tag{48}$$

$$T^-(n) = \sum_{i=n+1}^{N} \left(\frac{1}{(N-n)+wn} \right) \frac{N-i}{N-1} = \frac{(N-n)(N-n-1)}{2(N-1)[(N-n)+wn]}. \tag{49}$$

The corresponding rates are

$$r_N^+(n) = r\frac{wn(n-1)}{2[(N-n)+wn]} \tag{50}$$

$$r_N^-(n) = r\frac{(N-n)(N-n-1)}{2[(N-n)+wn]}, \tag{51}$$

which are transformed into the continuous rates

$$r^+(f) = rN\frac{wf^2}{2[1+(w-1)f]} \tag{52}$$

$$r^-(f) = rN\frac{f^2+2f-1}{2[1+(w-1)f]}. \tag{53}$$

The drift and diffusion terms are respectively

$$A(f) = rN\frac{(w-1)f^2+2f-1}{2[1+(w-1)f]} \tag{54}$$

$$B(f) = rN\frac{(w+1)f^2-2f+1}{2[1+(w-1)f]}. \tag{55}$$

The results presented in the main text are the numerical solutions of the probability and MFPT of fixations in Eqs 12 and 20 with these drift and diffusion terms.

In the main text, large fitness differences on the magnitude of 10-100 are explored. Such fitness differences are encountered in extreme situations, for instance in the scenario where one of the species is susceptible to a drug administered to the system. However, more moderate fitness differences that do not span orders of magnitude are also observed experimentally in less perturbed systems [8]. We show in Fig 2 how small fitness differences impact the probability of fixation for the fitter species and the MFPT of fixation for either species.

The area under the probability curve in the Moran model grows sharply in Fig 2A, whereas the sigmoidal probability in the spatial exclusion shifts very minimally. This indicates that moderate fitness differences do not confer large competitive advantages

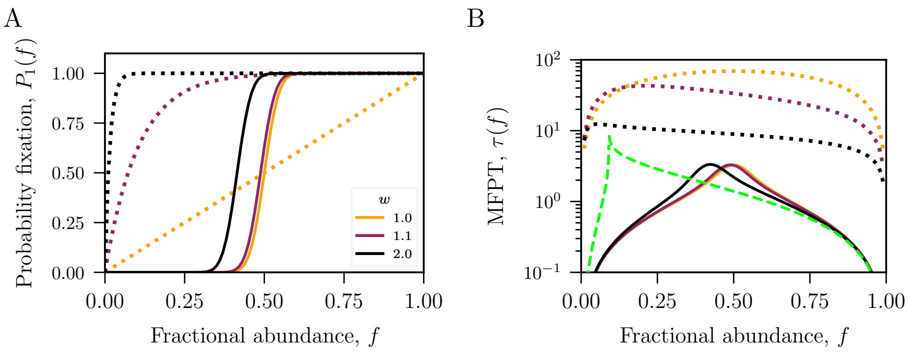

Fig 2. Competition outcomes for dynamics with small fitness differences. Fokker-Planck approximation for the fixation probabilities and the mean-first passage times (MFPTs) for both the Moran model (dotted lines) and the spatial exclusion model (solid lines) with selection ($w = 1, 1.1, 2.0$) and $N = 100$. The fractional abundance f is the abundance of the species with the selective advantage. **(A)** The fixation probabilities for both models with selection are shifted compared to neutral models, however the spatial model does not show much change. **(B)** In the spatial model, the maximum MFPT shifts only slightly with increases with fitness. The dashed lime line represents the shift of the MFPT when $w = 100$

to the fitter species in the spatial exclusion model as they do in the Moran model. Although the fitter species has a slight advantage in succeeding, the scale of the MFPT to fixation in the spatial exclusion model does not change much for most of fractional abundances, as seen in Fig 2B. We find that moderate fitness advantages do not impact the competitive dynamics in the spatial model as much as they do in a well-mixed model.

D.3 Comparing the master equation to Fokker-Planck

In Section A, the solution for the probability of fixation, Eq 6, and the MFPT to fixation, Eq 8, were presented for the discrete master equation formulation. These solutions require finding the inverse of a $N \times N$ matrix, which may be computationally intensive for large populations. Alternatively, the Fokker-Planck approach described in Section B provides a set of differential equations, Eqs 12 and 20, that are solved algebraically and/or numerically to obtain the probability and MFPT of fixation in the continuous approximation formulation. The continuous Fokker-Planck approximation differs only slightly from the exact discrete solution as shown in Fig 3.

As expected, differences between the exact solution and the Fokker-Planck approximation decrease as the system size N increases. For low N, the probability of fixation in the master equation formulation is steeper than the Fokker-Planck solution, as seen in Fig 3A. On the other hand, both approaches recover similar MFPT in Fig 3B. The similarity between master equation and Fokker-Planck formulations are maintained for various fitness differences, as seen in Figs 3C and 3D. The probabilities and the MFPT are consistent between the approaches for all fitness differences at $N = 100$.

D.4 MFPT conditioned on the success of one species

Results in the main text concerning the MFPT to fixation in the single boundary probelm refer to the MFPT to fixation by either species in the system. On the other hand, Eqs 8 and 20 describe the equations governing the MFPT to fixation conditioned on the success of one of the species. In other words, these equation can be solved to find the mean time

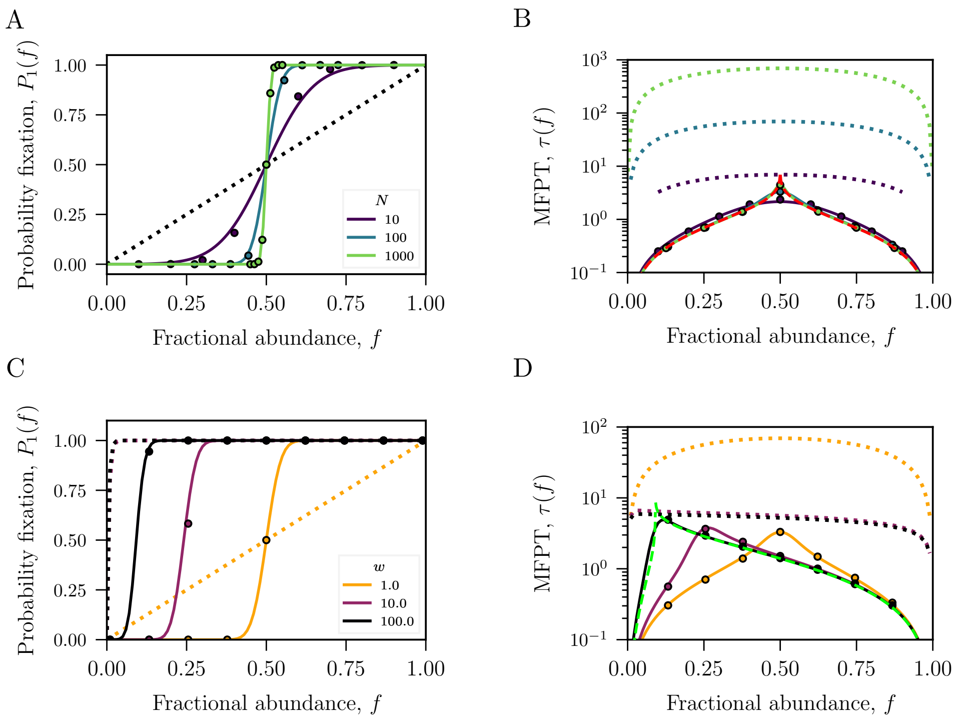

Fig 3. A comparison of the Fokker-Planck and master equation solutions with numerical solutions to the Fokker-Planck equation for the spatial model (solid lines) and Moran model (dotted lines) overlayed with numerical solutions to the master equation (scatter points) for the spatial model. **(A)** Probability of fixation and **(B)** MFPT to fixation for the neutral models as a function of fractional abundance. Plots for different population sizes (colors) show that differences between Fokker-Planck and the master equations decrease with higher population size N. At $N = 100$, **(C)** the probability of fixation and **(D)** the MFPT to fixation with fitness differences show negligible differences between ME and FP.

in which the species with fractional abundance f takes over the channel by reaching a final fractional abundance of $f_F = 1$. Conversely, the other species (which starts with a fractional abundance of $1 - f$) will successfully takeover the channel when $f_F = 0$. Numerical solutions for the fractional abundances solved in Eq 8 are shown in Fig 4.

The neutral competition with varying system sizes, N, is plotted in Fig 4A, showing the MFPT of successful fixations for the species with initial abundance f. For initial fractional abundances below the equiprobable takeover abundance at $f = 1/2$, the MFPT increases with N. In this regime, the probability that the species with fractional abundance f fixates is low. The fractional abundance of the species must increase beyond the equiprobable takeover abundance by drift before taking over deterministically; the diffusion increases the MFPT with N since the distance the boundary defined by the fractional abundance must diffuse to get to $f = 1/2$ increases with the system size, N. Conversely, initial fractional abundances above the equiprobable takeover abundance have a high likelihood of successfully taking over and we find that these MFPT conditioned on success are independent of N. Takeover by the species is approximately deterministic in this regime, resulting in MFPT that are independent of N as shown in the main text.

We show both the MFPT to successful fixation for the species with a fitness advantage and fractional abundance f, as well as the MFPT of successful fixation for the other,

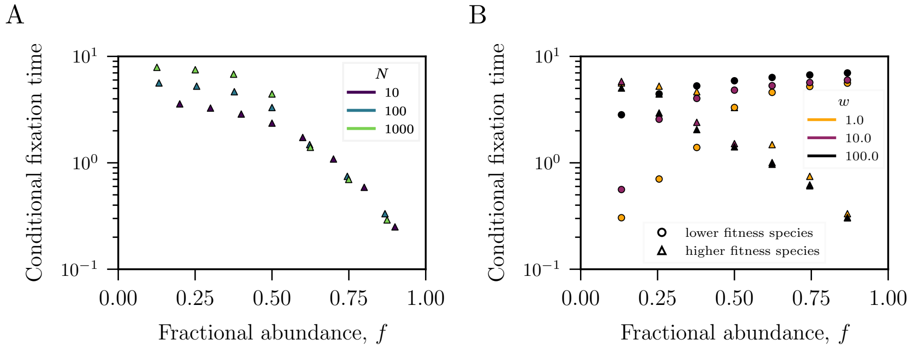

Fig 4. MFPT conditioned on the success of a species. The mean-first passage times (MFPTs) for the spatial exclusion model conditioned on the success of the species with a selective advantage (△ marker) and the species without a selective advantage (○ marker). The fractional abundance f is the abundance of the species with the selective advantage. **(A)** The MFPT to fixation conditioned on the success of the species with abundance f in the neutral case. The MFPT of fixation conditioned on the success of the other species (not plotted) is a reflection of these results about the axis $f = 1/2$. **(B)** With a selective advantage, the MFPT conditioned on the success of a species is no longer symmetric. Note that the fractional abundance of the less fit species is $1 - f$. The system size used for these results is $N = 100$

less fit species in Fig 4B. Unsurprisingly, when the fractional abundance, f, of the fitter species is below the equiprobable takeover abundance and the fractional abundance, $1-f$, of the other species is high, the MFPT for the high fitness species to fixate is larger than the MFPT for the low fitness species to fixate. In this state, successful fixation is deterministic for the low fitness species. The converse is true in the high abundance regime for the high fitness species, as its takeover is quick and roughly deterministic. Interestingly, the MFPT of successful fixation of each species intersect at a fractional abundance f above the equiprobable takeover abundance. In other words, the fitter species takes longer to successfully fixate than the less fit species at the equiprobable takeover abundance. Although both species are equally likely to take over the system at this state, the fitter species must go through more birth events than its competitor to successfully fixate given that the equiprobable takeover abundance is less than $f = 1/2$ for $s > 1$. These additional birth events increase the MFPT, resulting in longer fixation times for the species with a selective advantage.

Additionally, we find that the MFPT of successful fixation of the fitter species decrease with fitness difference and, conversely, the MFPT to successful fixation of the less fit species increases with fitness difference. This is to be expected, given that the fitter species is more frequently selected for birth events, causing fixations to be shorter for it and longer for the less fit competitor.

E Average probabilities and MFPT

One of the challenges faced in experimental settings is the inherent variability in initial conditions. In many real-world scenarios, it's often impractical or even impossible to precisely control or fix the initial frequency of this system. This variability arises from a range of factors such as environmental fluctuations, or other stochastic effects. To quantify the system's behavior across a spectrum of initial conditions, we can average different measurements over all possible initial conditions. By obtaining an aggregate

result through an averaging process, we can provide benchmark results that can be compared with experimental outcomes (such as in microfluidic realizations of these systems).

As outlined in the main text, the probability of fixation $P_N(n)$, the MFPT to fixation $\tau(n)$ and the directional MFPT of fixation $\tau_N(n)$ depend on the initial abundances of the species populations. An estimate of the outcome of the competition between two species is obtained by calculating these quantities averaged over all possible initial abundances. Assuming that all initial abundances are equally likely, the average probability is

$$\langle P_1 \rangle = \int_0^1 P_1(f) df. \tag{56}$$

Both $\langle \tau \rangle$ and $\langle \tau_N \rangle$ are calculated similarly and are plotted for various fitness difference and total population size in Fig 5.

The average probability to fixate increases with both the total population size and the (unsurprisingly) fitness difference, see Figs 5 and 5B. This probability in the spatial model converges to $1 - 1/(1 + \sqrt{w})$ as N increases in Fig 5A. The convergence is expected given that the probability distribution as a function of the fractional abundance converges to a step function whose inflection point is at $1/(1 + \sqrt{w})$ at high population size. Naturally, as the fitness of a species increases, the probability that the species succeeds in taking over the channel increases (Fig 5B). Additionally, we find that a species with a fitness advantage has a higher probability of fixating on average in the Moran model than in the spatial exclusion model. As discussed in the main text, this suggests that the competitive advantage conferred by fitness differences are attenuated in the spatial model.

In Fig 5C, we see that the average MFPT for both the Moran model and the spatial model increases as a function of total population size N. Intuitively, as the system size gets larger, the paths that lead to fixation require the species to push out more individuals on average which takes more time. However, the MFPT for the species with higher fitness to fixate decreases with system size (see Fig 5E), that is to say that successful fixations of the more fit species are more rapid even if there are more cells to push out. Consequently, channels take longer to fixate on average because the less fit species has very slow fixation times.

Interestingly, although on average the MFPT to fixation decreases with fitness increases (Fig 5D), the MFPT of fixation for the more fit species conversely increases on average (Fig 5F). This is counter-intuitive as it might be expected that a fitter species would have a more rapid takeover given the greater likelihood of being selected for birth events. However, as its fitness increases, a species can take over the channel from smaller initial fractional abundance from which more birth events are required to fill the channel. Therefore, higher fitness differences mean longer successful fixation paths for the fitter species as more competitors need to be pushed out of the channel on average.

F Invasion into a fixated channel

A homogeneous, single species population channel remains fixated until a species is reintroduced through a mutation or immigration event [9,10], at which point the invading and inhabitant species begin competing for fixation. Such an invasion event reintroducing a species may happen at any location in the channel, splitting the previously fixated population into two populations on either side of the channel. Assuming the invader species (with fitness difference w) does not enter the system at the edge of the channel, the channel in now segregated into "inhabitant-invader-inhabitant" populations. As the species grow, the state of the system is now defined by the locations of the 2 "inhabitant-invader" boundaries, where n is the number of inhabitant individuals on the left of the

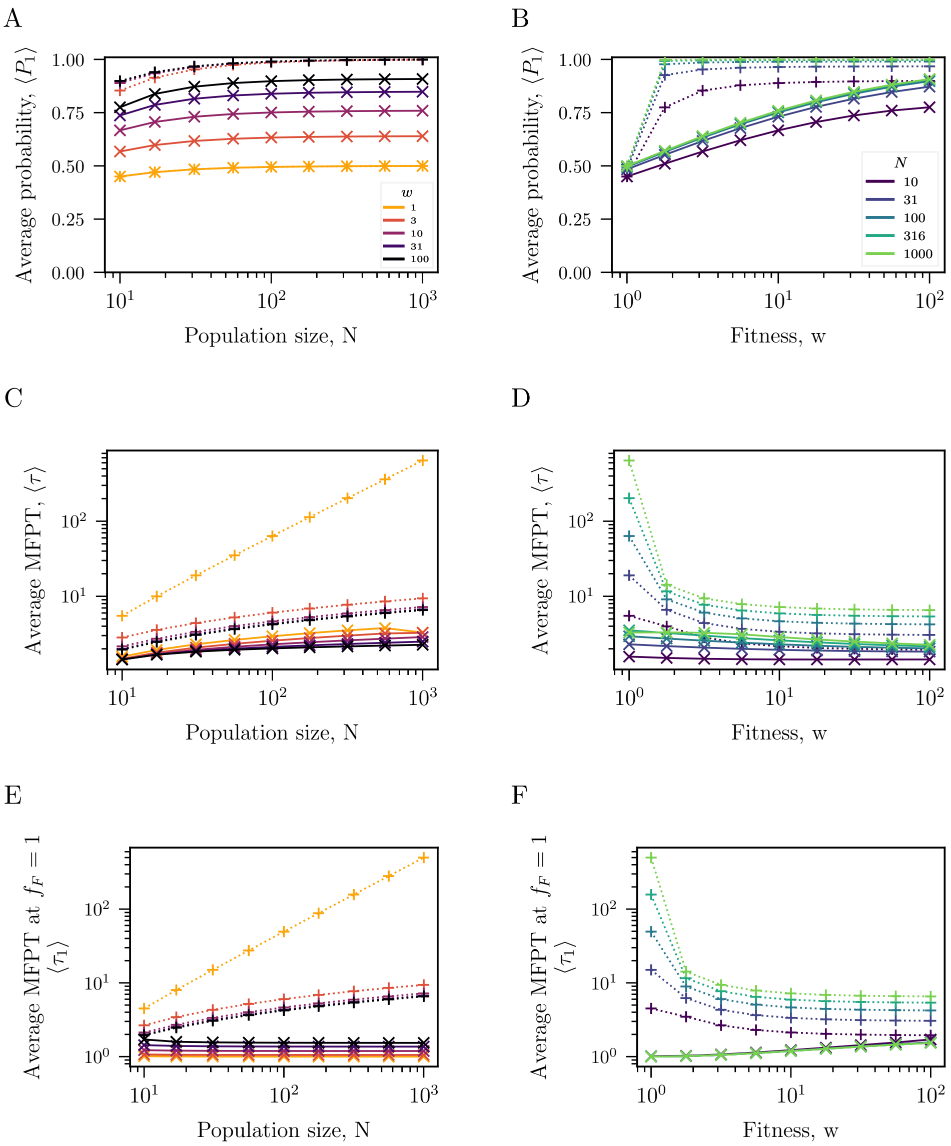

Fig 5. Outcomes of the competition averaged over all initial abundances. Numerical solutions to the Fokker-Planck equation for the spatial model ($\times$ connected with solid lines) and Moran model (+ connected with dotted lines). Average probability of the invader succeeding in fixating as a function of **(A)** population size and **(B)** fitness difference. Average MFPT of the fixation as a function of **(C)** population size and **(D)** fitness difference. Average MFPT of the more fit species fixating as a function of **(E)** population size and **(F)** fitness difference.

invader and m is the number of inhabitant individuals on the right of the invader. Given that the total population remains fixed, the number of invading individuals is $N-m-n$.

The dynamics of each boundary are similar to the dynamics of the single boundary in the one-dimensional spatial model of Section D: a boundary moves to the right (left) when an individual to its left (right) gives birth to the right (left). The probability of an invader individual with a fitness difference w being selected to undergo a birth event is $w/\left(n+m+w(N-m-n)\right)$ whereas a inhabitant individual is selected with

probability $1/\left(n+m+w(N-m-n)\right)$. Like the spatial model, the direction a cell pushes its progeny depends on the fraction of cells in the opposite direction from its movement. According to these rules, one, both or neither boundary can change location depending on which cell gives birth and in what direction the progeny is pushed.

For instance, if an invader species is selected for birth, either boundaries n or m will decrease by 1 as an inhabitant individual is pushed out to make room for the progeny of the invader. The rate at which either boundary decreases is

$$\begin{aligned} r(n,m\to n,m-1) &= N\sum_{i=n+1}^{N-m}\frac{w}{n+m+w(N-m-n)}\frac{i-1}{\Delta t(N-1)} \\ &= r\frac{(N-m)(N-m-1)}{2[n+m+w(N-m-n)]} - r\frac{n(n-1)}{2[n+m+w(N-m-n)]}, \quad (57) \\ r(n,m\to n-1,m) &= \sum_{i=m+1}^{N-n}\frac{w}{n+m+w(N-m-n)}\frac{i-1}{\Delta t(N-1)} \\ &= r\frac{(N-n)(N-n-1)}{2[n+m+w(N-m-n)]} - r\frac{m(m-1)}{2[n+m+w(N-m-n)]}. \quad (58) \end{aligned}$$

On the other hand, both boundaries move if an inhabitant individual on the right (left) of the invader gives birth and pushes its progeny to the left (right). The rates of both boundaries moving are

$$\begin{aligned} r(n,m\to n+1,m-1) &= \sum_{i=1}^{n}\frac{1}{n+m+w(N-m-n)}\frac{i-1}{\Delta t(N-1)} \\ &= r\frac{n(n-1)}{2[n+m+w(N-m-n)]}, \quad (59) \\ r(n,m\to n-1,m+1) &= \sum_{i=1}^{m}\frac{w}{n+m+w(N-m-n)}\frac{i-1}{\Delta t(N-1)} \\ &= r\frac{m(m-1)}{2[n+m+w(N-m-n)]}. \quad (60) \end{aligned}$$

Trivially, neither boundary changes if an inhabitant individual species on the right (or left) side of the channel gives birth to the right (or left).

Using the rates in Eqs 57 and 60, the two-dimensional master equation describing the evolution of the probability of the boundaries is

$$\begin{aligned} \partial_t p(n,m;t) &= r(n+1,m\to n,m)p(n+1,m;t) + r(n,m+1\to n,m)p(n,m+1;t) \\ &+ r(n+1,m-1\to n,m)p(n+1,m-1;t) + r(n-1,m+1\to n,m)p(n-1,m+1;t) \\ &- [r(n,m\to n+1,m-1) + r(n,m\to n-1,m+1) \\ &+ r(n,m\to n-1,m) + r(n,m\to n,m-1)]p(n,m;t). \quad (61) \end{aligned}$$

which can be expressed in matrix form as in Eq 3.

The boundaries at n and m fluctuate until either boundary reaches 0, at which point one of the 2 regions of the inhabitant species populations has been removed from the channel. From then on, only one boundary between an inhabitant population and the invader population is present in the system. This corresponds to the one-dimensional spatial dynamics described in Section D where two populations of species compete for fixation in the channel. Subsequently, the invading species successfully invades the channel if the absorbing state $(n,m)=(0,0)$ is reached, whereas the inhabitant species overcomes the invasion if either of the absorbing states $(0,N)$ or $(N,0)$ are reached.

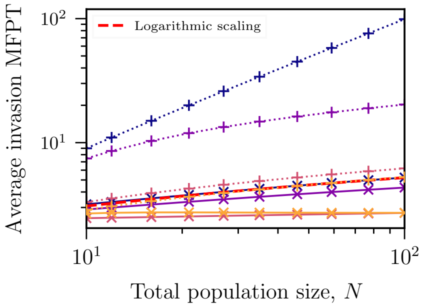

Fig 6. Invasion into a fixated channel, continued. Numerical solutions to the Master equation for the spatial model (× connected with solid lines) and Moran model (+ connected with dotted lines). The average MFPT of a successful invasion in the spatial model increases as population size increases, except at high fitness.

The probability and MFPT of the outcome for an invasion event can be numerically calculated by solving Eqs 6 and 8.

In a continuous state space, the invasion starting from an initial position $(n, N-n-1)$ is defined as an invasion at location x and the probability of success of this invasion we denote $P_{\text{inv}}(x)$. The MFPT of an invasion at a location x conditioned on success is $\tau_{\text{inv}}(x)$. In the main text, we describe the average invasion probability, which is

$$\langle P_{\text{inv}} \rangle_x = \int_0^1 dx P_{\text{inv}}(x) p_{\text{init}}(x) \tag{62}$$

where $p_{\text{init}}(x)$ is the probability of the initial invasion being at location x, which we assume is uniform. The average of the invasion MFPT conditioned on the success of the invasion, is

$$\langle \tau_{\text{inv}} \rangle_x = \int_0^1 dx \tau_{\text{inv}}(x) \frac{P_{\text{inv}}(x)}{\int_0^1 dx P_{\text{inv}}(x)}. \tag{63}$$

In the main text, we show that the average of the invasion MFPT conditioned on the success of the invasion increases (albeit slowly) with population size, N. The mean successful invasion times exhibit non-monotonic behavior at very high fitness levels, where the average invasion duration increases with rising fitness, see Fig 6. This phenomenon can be attributed to the fact that as fitness levels increase, a larger number of invasion locations away from the center of the lane result in successful invasions. When an invasion starts from a location further from the center, the invader population must generate more offspring to reach the farthest end of the lane, which consequently takes more time (increasing logarithmically with system size at the equiprobable abundance). As a result, when we average the durations of successful invasions over a greater number of invasion locations, we observe generally longer times. This behavior results in a different scaling of the MFPT, which begins to decrease with increasing system size at large fitness differences.

F.1 Invasions from the edge of the channel

To compare a nominal invasion in the spatial model to the most similar invasion scenario in the Moran model, we investigate an invasion of two individuals ($n = 2$) on the edge of the channel. Starting with 2 individuals is necessary because an invasion at the edge of the channel in the spatial model cannot successfully takeover if it is undertaken by a

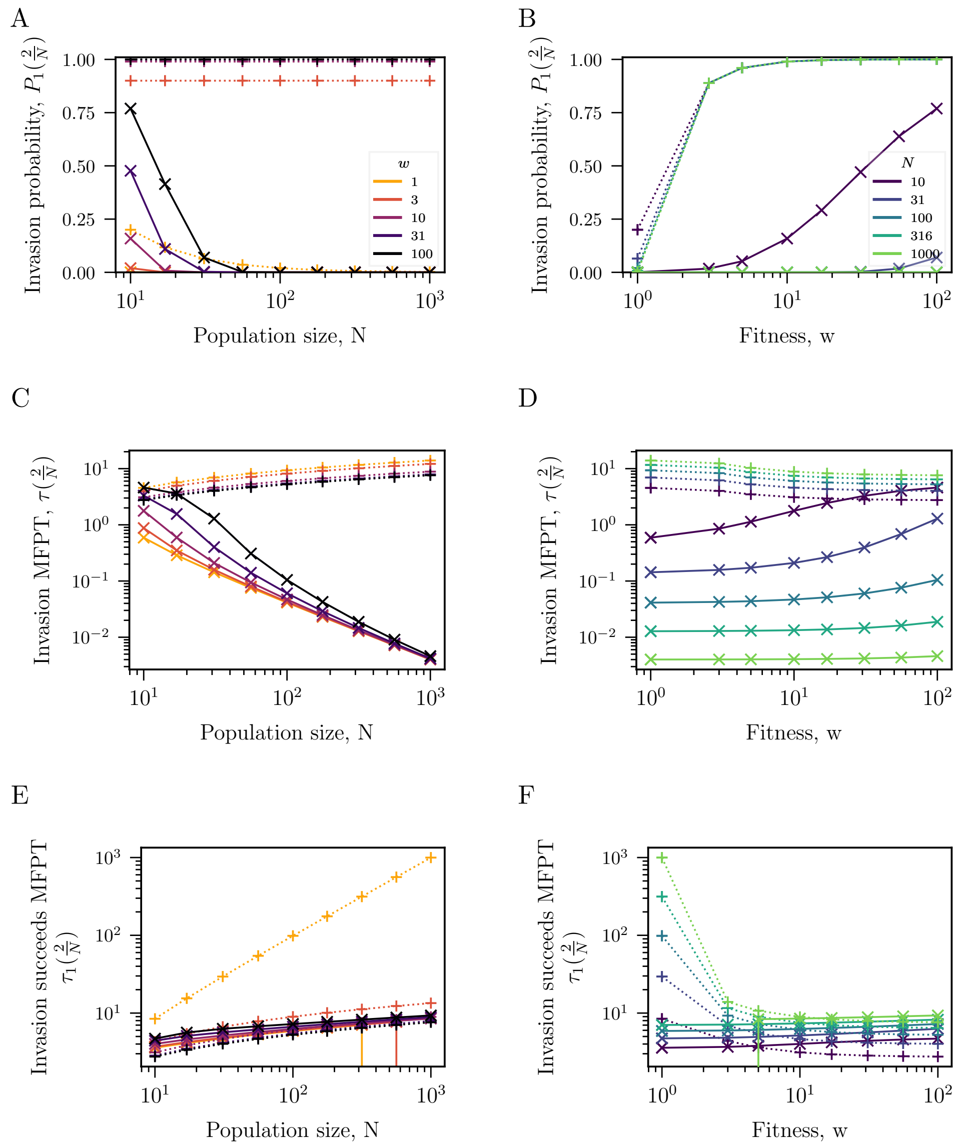

Fig 7. Outcome of an invasion of 2 individuals from the edge of the channel. Numerical solutions to the Fokker-Planck equation for the spatial model (× connected with solid lines) and Moran model (+ connected with dotted lines). The probability of the invader succeeding in fixating as a function of **(A)** population size and **(B)** fitness difference. The MFPT of fixation as a function of **(C)** population size and (D) fitness difference. The directional MFPT of the invader succeeding in fixating as a function of **(E)** population size and **(F)** fitness difference. Numerical instabilities in **E** and **F** are a consequence of the low probability of fixation at low fitness ($w = 1, 3$ in **E**) and high population size ($N = 1000$ in **F**.

single individual. This is an artifact of our model since $r_N^+(n = 1) = 0$ in Eq 51, i.e. a species with a single individual at the edge of the channel cannot grow in numbers. When a cell gives birth, the progeny is assuredly pushed in the direction of least resistance out of the channel.

The results of the nominal invasion ($n = 2$) in the spatial exclusion model and Moran

model are presented in Fig 7.

The probability of a nominal invasion succeeding in the Moran model is much larger than in the spatial model, see Fig 7A. Although the probability of fixation in the neutral Moran model slowly decreases with population size N, the probability of fixation remains high and relatively constant for large fitness differences at all population size, ensuring successful invasions for any length channel. Conversely the probability of successful fixation for nominal invasion in the spatial model decreases rapidly as population size increases.

Unsurprisingly, fitness increases lead to higher probability of fixation for both models, with fitter species in the Moran model benefiting more from their selective advantage, see Fig 7B.

In Fig 7C, the spatial model exhibits shorter MFPT to fixation as the population size increases. The MFPT of fixation are shorter since the inhabitant species are more abundant as the channel is longer and more individuals from the inhabitant species are present to push out the few invaders quickly. Conversely, the MFPT for the invader to successfully fixate grows with the system size (Fig 7E) since longer channels mean that the invader needs to push out more inhabitants to takeover.

In Figs 7D and 7F, the MFPTs of fixation are plotted as a function of fitness. Both the overall MFPT and directional MFPT increase with fitness difference in the spatial model; higher fitness differences cause the competition between the species to be prolonged. This is because the invader species is selected more frequently for birth events, providing the species more time to gain in abundance before the inhabitant species is able to eventually grow and push it out.

G The heuristic asymptotic MFPT approximation

In this section, we derive a heuristic large N scaling of the MFPT, As given in Eq 18, the exact integral form of the MFPT for our one-dimensional stochastic process is

$$\tau(f) = 2N^2 \frac{1}{\int_0^1 dy e^{U(y)}} \left(\left(\int_0^f dy e^{U(y)} \right) \int_f^1 dy' e^{U(y')} \int_0^{y'} dz \frac{e^{-U(z)}}{B(z)} - \left(\int_f^1 dy e^{U(y)} \right) \int_0^f dy' e^{U(y')} \int_0^{y'} dz \frac{e^{-U(z)}}{B(z)} \right) \quad (64)$$

where $U(x)$ is defined in Eq 19. Using the definitions of the drift and diffusion terms of the spatial model (Eqs 54, 55 and 19) we get for the effective potential:

$$U(f) = -2N \frac{2w \log\left[(w+1)f^2 - 2f + 1\right] + (w^2-1)f - 2\sqrt{w}(w-1)\arctan\left[\frac{(w+1)f-1}{\sqrt{w}}\right]}{(w+1)^2}. \quad (65)$$

As shown in the main text and in Fig 8A, this potential is a concave function with a peak at $f_{\mathrm{eq}} = 1/(1+\sqrt{w})$.

Using Watson's Lemma, we expand the expression in the first summand around the maximum of $-U(z)$ at $z = 0$. In Equation 72, we used Watson's Lemma, [11,12]. Similar to the method of steepest descent, Watson's Lemma is employed here to approximate the integral at the point where the integrand dominates the expression near the interval

edge. This yields

$$
\begin{aligned}
S_1 &= \lim_{N\to\infty} 2N^2 \frac{\int_0^f dye^{U(y)}}{\int_0^1 dye^{U(y)}} \int_f^1 dy' e^{U(y')} \int_0^{y'} dz \frac{e^{-U(z)}}{B(z)} \\
&\approx \lim_{N\to\infty} 2N^2 \frac{\int_0^f dye^{U(y)}}{\int_0^1 dye^{U(y)}} \int_f^1 dy' e^{U(y')} e^{-U(0)} \int_0^{y'} \frac{e^{-U'(0)(z-0)}}{B(z)} \\
&= \lim_{N\to\infty} 2N^2 \frac{\int_0^f dye^{U(y)}}{\int_0^1 dye^{U(y)}} \int_f^1 dy' e^{U(y')} e^{-U(0)} \Bigg(\left[-\frac{e^{-U'(0)(z)}}{U'(0)B(z)} \right]_0^{y'} \\
&\qquad\qquad + \int_0^{y'} \frac{d}{dt} dz \left(\frac{1}{B(z)U'(0)} \right) e^{-U(0)z} \Bigg).
\end{aligned} \tag{66}
$$

Because $U \propto N^{-1}$, it is clear with further integration by parts that the integral in the last parenthesis is $\mathcal{O}(N^{-2})$ and can be ignored in the asymptotic expansion. This leaves us with a sum of two integrals,

$$
S_1 \approx 2N^2 \frac{\int_0^f dye^{U(y)}}{\int_0^1 dye^{U(y)}} \left(\frac{e^{-U(0)}}{U'(0)B(0)} \int_f^1 dy' e^{U(y')} - \frac{e^{-U(0)}}{U'(0)} \int_f^1 dy' \frac{e^{U(y')-U'(0)y'}}{B(y')} \right). \tag{67}
$$

We can similarly expand the second term in Eq 64 using integration by parts

$$
\begin{aligned}
S_2 &= -\lim_{N\to\infty} 2N^2 \frac{\int_f^1 dye^{U(y)}}{\int_0^1 dye^{U(y)}} \int_0^f dy' e^{U(y')} \int_0^{y'} dz \frac{e^{-U(z)}}{B(z)} \\
&= \lim_{N\to\infty} 2N^2 \frac{\int_f^1 dye^{U(y)}}{\int_0^1 dye^{U(y)}} \int_0^f dy' e^{U(y')} \left(\left[\frac{e^{-U(z)}}{U'(z)B(z)} \right]_0^{y'} - \int_0^{y'} \left(\frac{1}{U'(z)B(z)} \right)' e^{-U(z)} dz \right),
\end{aligned} \tag{68}
$$

Again, the integral term in the parenthesis is dropped because it is of order $\mathcal{O}(N^{-2})$. Eq 68 can be written as

$$
S_2 \approx \lim_{N\to\infty} 2N^2 \frac{\int_f^1 dye^{U(y)}}{\int_0^1 dye^{U(y)}} \left(\int_0^f dy' e^{U(y')} \frac{e^{-U(y')}}{U'(y')B(y')} - \frac{e^{-U(0)}}{U'(0)B(0)} \int_0^f dy' e^{U(y')} \right) \tag{69}
$$

After some algebra leading to cancellation of terms in S_1 and S_2, we are left with

$$
\begin{aligned}
\tau(f) = S_1 + S_2 \approx \lim_{N\to\infty} 2N^2 \Bigg[& \frac{\int_f^1 dye^{U(y)}}{\int_0^1 dye^{U(y)}} \int_0^f dy' e^{U(y')} \frac{e^{-U(y')}}{U'(y')B(y')} \\
& - \frac{\int_0^f dye^{U(y)}}{\int_0^1 dye^{U(y)}} \frac{e^{-U(0)}}{U'(0)} \int_f^1 dy' \frac{e^{U(y')-U'(0)y'}}{B(y')} \Bigg]
\end{aligned} \tag{70}
$$

Below, we derive approximate solutions for Eq 70 in two regimes: when the initial relative abundance is close to the equiprobable abundance, $f \approx f_{\text{eq}}$, and when it is not ($f \ll f_{\text{eq}}$ or $f \gg f_{\text{eq}}$).

Case 1: $f \ll f_{\mathrm{eq}}$ or $f \gg f_{\mathrm{eq}}$

We show the solutions only for the case $f \ll f_{\mathrm{eq}}$ but equivalenty results are obtained for $f \gg f_{\mathrm{eq}}$. First, since the potential is a concave function, we can calculate the normalization factors in the denominator of Equation 70 using the saddle-point approximation using asymptotic methods [13]. Considering that $U(f)$ is proportional to N, we can approximate the integral as follows:

$$R_d = \lim_{N\to\infty} \int_0^1 e^{U(f)} dx \approx \sqrt{\frac{2\pi}{|U''(f_{\mathrm{eq}})|}} e^{U(f_{\mathrm{eq}})}. \tag{71}$$

Further, note that the summand S_1 is proportional to $R_1 = \lim_{N\to\infty} \int_0^f dy e^{U(y)}$, and S_2 is proportional to $R_2 = \lim_{N\to\infty} \int_f^1 dy e^{U(y)}$. Each of the integrals R_1 and R_2 can be solved in the asymptotic limit,

$$R_1 = \lim_{N\to\infty} \int_0^f dy e^{U(y)} \approx \frac{1}{U'(f)} e^{U(f)} \tag{72}$$

$$R_2 = \lim_{N\to\infty} \int_f^1 dy e^{U(y)} \approx \sqrt{\frac{2\pi}{|U''(f_{\mathrm{eq}})|}} e^{U(f_{\mathrm{eq}})} \tag{73}$$

In Equation 72, we used Watson's Lemma, [11,12] expanding around the maximum of the integrand that for large N occurs at the edge point f.

In the large N limit for $f \ll f_{\mathrm{eq}}$, Eq 70 is dominated by the R_2 term In this limit, Eq 70 is reduced to

$$\begin{aligned}\lim_{N\to\infty} \tau(f) &\approx 2N^2 \frac{\int_f^1 dy e^{U(y)}}{\int_0^1 dy e^{U(y)}} \int_0^f dy' e^{U(y')} \frac{e^{-U(y')}}{U'(y')B(y')} \\ &= -N \int_0^f dy' \frac{1}{A(y')} \\ &= -\log \left| (w-1)f^2 + 2f - 1 \right|. \end{aligned} \tag{74}$$

As discussed in the main text, Eq 74 is the deterministic time $\tau_{\mathrm{det}}(f)$ for a variable to reach 0 from f under the influence of a deterministic drift force $A(f)$. The system performs predominantly deterministic motion down the potential gradient due to the skewed collective growth in the regime $f \ll f_{\mathrm{eq}}$ (or the symmetric regime $f \gg f_{\mathrm{eq}}$).

Case 2: $f \approx f_{\mathrm{eq}}$

When the relative abundance approaches the equiprobable abundance, the terms in Eq 70 proportional to R_1 and R_2 are of the same order of magnitude.% with $M \to 1$ in $R_2(f) = MR_1(f)$. In this limit, the deterministic solution no longer dominates the time to fixation. Heuristically, we define the boundary abundance where the deterministic solution stops being a good approximation, f_t^-, as the point where $R_2(f_t^-) = R_1(f_t^-)$. This assumption is justified *a posteriori* by a comparison with the full numerical solution. Assuming that the abundance f_t^- is close to the peak of the potential, we find that

$$\lim_{N\to\infty} R_1(f_t^-) \approx \frac{1}{U'(f_t^-)} e^{U(f_t^-)} \approx \frac{1}{U'(f_t^-)} e^{U(f_{\mathrm{eq}})} \tag{75}$$

$$\lim_{N\to\infty} R_2(f_t^-) \approx \sqrt{\frac{\pi}{2|U''(f_{\mathrm{eq}})|}} e^{U(f_{\mathrm{eq}})} \tag{76}$$

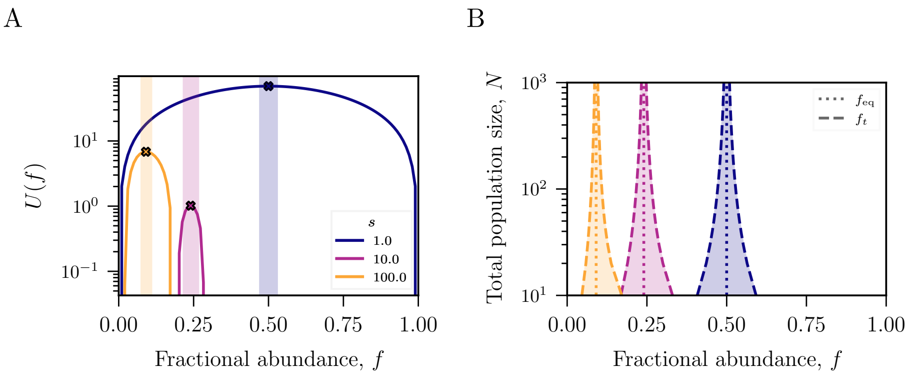

Fig 8. Regions of dominant stochastic behaviour for different populations (shaded). **(A)** The effective potential (lines) with regions of dominant stochastic behaviour (shaded) around the peak of the potential (scatter point) for $N = 100$. The region decreases for higher fitness. **(B)** The region of relative fractional abundances where stochastic dynamics dominate varies by population size. As population size increases, the region shrinks for all fitness differences.

using the saddle-point method and Watson's Lemma as before. In Eq 75, $e^{U(f_{\text{eq}})} \approx e^{U(f_t^-)}$ given that f_t^- is close to the peak and $U(f) \propto N$. Additionally, 1/2 appears in Eq 76 compared to Eq 73.

The deterministic boundary abundance, f_t^-, is thus defined as the solution to

$$R_1(f_t^-) = R_2(f_t^-) \quad \rightarrow \quad U'(f_t^-) = \sqrt{\frac{2U''(f_{\text{eq}})}{\pi}}.$$

This equation has two solutions, the first of which is

$$f_t^- = \frac{1 + \sqrt{w\left(\frac{\pi N \sqrt{w}}{(1+\sqrt{w})^2} - 1\right)} - \frac{\sqrt{\pi N \sqrt{w}}}{1+\sqrt{w}}}{\frac{\sqrt{\pi N \sqrt{w}}}{1+\sqrt{w}}(w-1) + (w+1)}. \tag{77}$$

The second solution corresponds to the other boundary point $f_t^+ > f_{\text{eq}}$ which is shown with the left boundary in Fig 8. As N increases, the deterministic abundance boundaries, f_t^- and f_t^+, get closer to the peak of the effective potential at $f_{\text{eq}} = 1/(1+\sqrt{w})$ (see Fig 8B), widening the regions of abundances, $[0, f_t^-]$ and $[f_t^+, 1]$, in which the MFPT behaves deterministically.

The mean time to fixation from within the this region can be approximately written as a weighted sum of the time to diffuse from $f \rightarrow f_t^{\pm}$ and the time to determiniscally drift from $f_t^{\pm}$ to the extinction abundances, $f = 0$ or $f = 1$.

$$\tau_a(f) = p_{\text{dif}}(f \rightarrow f_t^+)\left(\tau_{\text{dif}}(f, f_t^+) + \tau_{\text{det}}(f_t^+)\right) + p_{\text{dif}}(f \rightarrow f_t^-)\left(\tau_{\text{dif}}(f, f_t^-) + \tau_{\text{det}}(f_t^-)\right), \tag{78}$$

where $p_{\text{dif}}(f \rightarrow f_t^{+/-})$ is the probability that the fractional abundance f diffuses to one of the boundaries at $f_t^{+/-}$.

For illustrative purposes, we examine the fixation time starting at $f = f_{\text{eq}}$ given that there is an equal probability to diffuse to either bound from this position. Using the deterministic boundary abundances in Eq 77 as the abundances in Eq 74, and noting

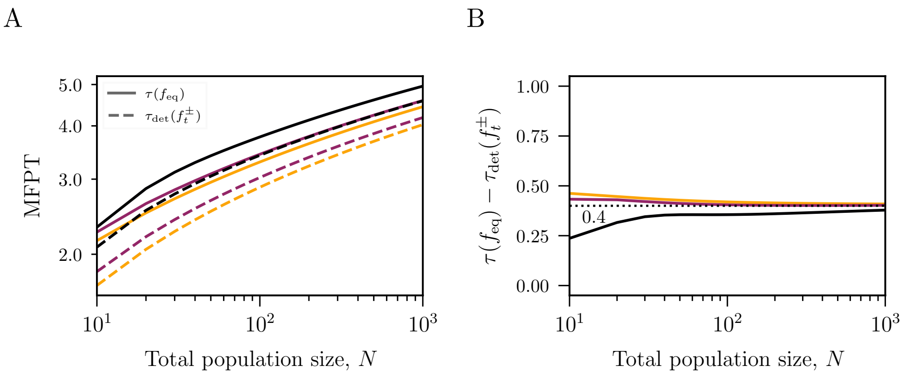

Fig 9. Heuristic approximation of the maximal MFPT. **(A)** The maximal MFPT to fixation $\tau(f_{\mathrm{eq}})$ is marginally larger than the time to fixation from the boundary of the deterministic region $\tau_{\mathrm{det}}(f_t^{\pm})$, however both exhibit logistic growth in N. **(B)** The difference between these two times converges to a constant for various fitness differences. This constant is roughly 0.4.

that the deterministic time $\tau(f_t^-)$ in Eq 74 scales as $\mathcal{O}(\log N)$ for large N, we recover asymptotic behaviour discussed in the main text

$$\tau_a(f_{\mathrm{eq}}) \sim \frac{1}{2}\log(\pi N) + \frac{1}{2}\left(\tau_{\mathrm{dif}}\left(f_{\mathrm{eq}}, x_t^-\right) + \tau_{\mathrm{dif}}(f_{\mathrm{eq}}, x_t^+)\right), \tag{79}$$

see Fig 9A. The difference between the MFPT from the peak of the effective potential and the deterministic time to fixation from either boundary converges to 0.4 as N increases, see Fig 9B. This suggests that the time of the diffusion of the boundary near the peak of the effective potential to the deterministic boundary transition points is constant in time. Further analysis is needed to confirm the value of the constant or determine if there is a slight dependence of fitness difference asymptotically in N.

References

1. Nisbet RM, Gurney WSC. Modelling Fluctuating Populations. Toronto: John Wiley & Sons; 1982.

2. Gardiner CW. Handbook of Stochastic Methods. 3rd ed. New York: Springer; 2004.

3. Karlin S. A first course in stochastic processes. Academic press; 2014.

4. Zhang J. Backward stochastic differential equations. In: Backward Stochastic Differential Equations. Springer; 2017. p. 79–99.

5. Iyer-Biswas S, Zilman A. First-Passage Processes in Cellular Biology. Advances in Chemical Physics. 2016;160:261–306. doi:10.1002/9781119165156.ch5.

6. Moran PAP. The statistical process of evolutionary theory. Clarendon Press; 1962.

7. Nowak MA. Evolutionary dynamics: exploring the equations of life. Harvard university press; 2006.

8. Kassen R, Bataillon T. Distribution of fitness effects among beneficial mutations before selection in experimental populations of bacteria. Nature genetics. 2006;38(4):484–488.

9. Giraud A, Matic I, Tenaillon O, Clara A, Radman M, Fons M, et al. Costs and benefits of high mutation rates: adaptive evolution of bacteria in the mouse gut. science. 2001;291(5513):2606–2608.

10. Aires T, Serrão EA, Kendrick G, Duarte CM, Arnaud-Haond S. Invasion is a community affair: clandestine followers in the bacterial community associated to green algae, Caulerpa racemosa, track the invasion source. PLoS One. 2013;8(7):e68429.

11. Zalik RA. The method of laplace and watson's lemma. Journal of Concrete and Applicable Mathematics. 2012;10.

12. Temme NM. Asymptotic methods for integrals. vol. 6. World Scientific; 2014.

13. Erdélyi A. Asymptotic Expansions, Dover. New York. 1956;.
