# Supplementary material for: Spatial exclusion leads to “tug-of-war” ecological dynamics between competing species within microchannels
Source: PLoS Comput Biol. 2023 Dec 1;19(12):e1010868. doi: 10.1371/journal.pcbi.1010868 (PMC10718426; doi:10.1371/journal.pcbi.1010868)
Supplement: Attachment — Submitted filename: Spatial-Exculsion-ReviewerResponse-Resubmission.pdf [file pcbi.1010868.s002.pdf]

Dear Dr. Weissman and Dr. O'Dwyer,

We appreciate the enthusiasm and the constructive criticism provided by the reviewers. With this resubmission, we have significantly revised our manuscript making every effort to address the comments/concerns raised by the referees. We hope these revisions satisfies the editors' and the reviewers' comments and the paper is acceptable for publication in PLoS Computational Biology.

Response to Reviewer #1:

1) In the abstract, there should be some mention that there is no death, but loss via the open ends of the channel. For instance, complement the sentence ending by "within a one-dimensional open microchannel." with something like ", in which cell loss happens only at the channel ends.". In the author summary, there is no mention that this is a 1-dimensional result, which I think is important.

We thank the reviewer for this suggestion and revised the manuscript accordingly: "*Informed by recent experimental studies of cellular competition in confined spaces, we extend the Moran model to explicitly incorporate spatial exclusion through mechanical interactions among cells within a one-dimensional, open microchannel, in which cell loss happens only at the channel ends.*"

2) There is a vocabulary confusion between the fixation time (of either types), and the fixation time conditioned on the mutant success. The MFPT is defined as the time of absorption in a state with either only all WT or all mutant. Most figures are showing this MFPT; only a few figures in appendix show the fixation time conditioned on mutant success. Yet, it seems that most commentaries are confusing these two different quantities. In the abstract for instance, the "the fixation times increase as the relative fitness differences between species increase." gives the impression that the fixation of a mutant goes slower with a higher fitness, whereas actually this is the time for fixation of either the WT or the mutant, and as the probability for the mutant to fix increases, the time to fixation for either types increases, because the time for fixation of the mutant is longer than the time of fixation of the WT. There are only 2 figures, panels 3F and 6F in appendix, that show, both in case when the mutants are on one end of the channel, there is indeed a very weak increase with fitness of the fixation time conditioned on success; but in a context in which the fixation probability increases greatly with fitness, so that actually in the few cases in which the mutant fixes at low fitness, the process has been very lucky, and thus quick. But my supposition is that for the case of a mutant arriving anywhere in the channel, by migration from a neighboring channel, the conclusion would be reversed. In this case, the successful invasions are from the middle of the channel, and the larger the fitness, the faster they would occur (but that would need to be checked).

We thank the reviewer for the comments and regret the confusion. In the first two subsections of the Results section, which discusses the case with one boundary between the two species within the channel model, we refer to unconditional fixation times simply as "fixation times"; the focus of the discussion is on the system fixation of either species rather than the fixation of a particular species. In particular, in Figure 3 of the main text, we are indeed showing unconditional fixation times (of either of the species) and find that the maximal fixation times increase as fitness difference increases. We rephrased the sentence in the abstract to better reflect this finding.

To address the reviewer's concern, we also added a Supplementary Figure S4 and displaying conditional fixation times for the low and high fitness species, showing how the conditional fixation times as a function of the relative abundance change with fitness difference. As suggested by the reviewer, the mutant (higher fitness) species takes longer to fixate starting at the equiprobable takeover abundance than the less fit species. As the probability of the "mutant" (higher fitness species) to fixate increases (i.e., abundance of the mutant increases beyond the equiprobable takeover abundance, the MFPT conditioned on the fixation of the higher fitness species decreases. Additional results are described in the new Supplementary section 4.4.

In contrast with the unconditioned times presented in the previous sections, the invasion times (starting with one invader cell, so that there are two inter-species boundaries) in the third subsection of the Results section are always conditioned on the success of the invader. We have clarified this everywhere in the text. In particular, in Figure 4 of the main text, we show the conditional invader fixation times for the two-boundary case, conditioned on the invader success. We have further emphasized this in the text.

As noted by the reviewer, Supplementary Figure 3F shows conditional fixation times for the one-boundary problem (same as Figure 3 in the main text) averaged over all initial positions. These conditional averaged fixation times increase with fitness difference increases. Similarly, the conditional fixation time of invasions with the invader starting at the edge of the channel increases in Supplementary Figure 6F. Both these figures as well as Figure 4D in the main text support our claim that the average fixation time conditioned on the invader fixating increases with fitness.

We have clarified the terminology throughout the text and referenced the results in the Supplementary Material. We hope this addresses the Reviewer's concerns.

3) In the abstract also: "Surprisingly, in the spatial exclusion model, successful fixations occur on average more rapidly in longer channels": actually in the whole manuscript only unconditional fixation time is shown, except for panels 3E, 3F, 6E and 6F in appendix, and for 6E and 6F, they actually show that in the model considered in these figures, the fixation time conditioned on mutant success does increase with N.

As in the previous comment, we have clarified the terminology, and added a figure (Fig S4) in the Supplementary Material.

4) from line 355: "Additionally, we find that the average MFPT of a successful invasion in the spatial model increases with fitness advantage (...) This is contrary to the intuition that higher selective advantage should accelerate the fixation of the invasive species because the fitter invader grows more rapidly.(...)" Actually what increases with the fitness is the average unconditional MFPT, thus actually the main effect is likely that at higher fitness, the mutant is more likely to fix, and fixation of a mutant takes longer than loss of the mutant.

In this section we consider fixation times conditioned on the success of the invader starting with one invading cell, which we refer to simply as "invasion time". We regret the confusion and have clarified the terminology by specifying that these are fixation times conditioned on the success of the invader. For instance, in Line 318: "*Whereas the MFPT presented in previous sections were the fixation times for either species to fixate, the invasion fixation time refers to the MFPT conditioned on the success of the invader*". We also remind the readers of this distinction in Line 344: "*To provide a global measure of invasion*

success, we calculate the average invasion probability and the average MFPT conditioned on the success of the invasion, averaged over all possible initial locations of the invasion event".

We hope this addresses the reviewer's concern.

5) section titled "Invasions are less likely to succeed due to spatial exclusion, but succeed on shorter timescales" whereas almost all results are on unconditional MFPT, and the only cases where MFPT conditioned on invasion success is shown, sup figures 3EF 6EF, there are actually in 6EF parameters for which the success MFPT is longer in the spatial model than in the well mixed model.

In this section (with two inter-species boundaries) the invasion times are always conditioned on the success of the invader. We regret the confusion, and have further clarified this in the text, in the Introduction, and the abstract. We also changed the section title to "*Invasions in spatial models are less likely to succeed and succeed on shorter timescales on average than in well-mixed models*" to better reflect its content.

6) from line 447 "Furthermore, competition between species with higher fitness differences show longer fixation times despite the fact that one of the species has a greater likelihood of dominating the channel. The slower fixations are owing to slower diffusion relative to drift around the point of equiprobable abundance." As said before, not proved. "This means that not only must a species preserve a potentially more resource intensive strategy to gain an advantage, but it must sustain this strategy for longer to ensure it dominates. Thus, the overall competitive advantage provided by an increase in fitness must be weighed against these two competing effects." Given the discussion is on the unconditional MFPT, not the MFPT conditioned on success, the last part does not make sense.

We have changed the wording of this passage to better reflect the predictions of the model. The time of fixation conditioned on success minimally decreases with fitness increases as is now explained in the Supplementary. Accordingly, the cells must weigh the benefit of increased probability of success with the cost of maintaining a resource-intensive strategy for a long time. The updated paragraph is at Line 470.

What's more, the unconditional time to fixate at the equiprobable takeover abundance increases with fitness difference. At this abundance, the fitter species has the same probability to successfully takeover as the less fit species, yet the competition now lasts longer than at lower fitness differences. The populations must make a decision on the strategy to undertake based on the trade-off between higher success in the chamber and shorter competition times.

7) around line 461: "the mean invasion times averaged over the initial channel location decrease with system size. These fixation times accelerate in longer channel because successful invasions with larger population sizes are reliant on the invader species remaining near the center of the channel, which fixates more rapidly. Consequently, successful invasions in longer channels happen less frequently, but those that are successful are more rapid on average." There is no figure with MFPT conditioned on success with the model with invasion anywhere in the channel. So no basis for making such a claim.

We regret the confusion of the fixation times in this section of the main text. We have specified that the fixation times that are considered in this section are all conditioned on the success of the invader.

**Other notations that are confusing:*

8) Usually s is used for the increase in fitness, not for the fitness itself (usually the fitness is noted as w=1+s). It is defined clearly in the manuscript, so it's ok, but it is a bit unusual notation.

We thank reviewer for this comment. We have chosen to follow the same convention as in *Nowak, Martin A. Evolutionary dynamics: exploring the equations of life. Harvard university press, 2006* and *Constable, George WA, and Alan J. McKane. "Stationary solutions for metapopulation moran models with mutation and selection." Physical Review E 91, no. 3 (2015): 032711.* We have added citations to these papers.

9) the "abundance at which both species are equally likely to take over the channel" is called "equiprobable abundance", which is confusing if the reader has missed the definition. It's a bit longer to write, but "equiprobable takeover abundance" would be clearer.

We have added this suggestion of the reviewer into our manuscript.

10) sometimes x represents the frequency of mutants, sometimes it represents the position in the channel. It would be much clearer to use one notation for frequency, and another for space, consistently in the whole manuscript.

As per the suggestion of the reviewer, we have clarified the notation in the main text and the Supplementary by using x to represent the position in the channel and f to represent the relative abundance (frequency) of the species with a fitness advantage.

**few things that should really be corrected, either statements that are not supported by the data, or equations (possibly typos):*

11) line 295 "In other words, the MFPT away from the equiprobable abundance will asymptotically decrease with fitness differences." Figure 3B: this is ok on the right side of xmax, but on the left side of xmax, it's actually the reverse, at a given x, the MFPT increases with fitness differences.

We have removed this statement from the manuscript, as it is misleading.

12) equation (12): to be equal to the right hand side, the left hand side should be -N exp(-U(x)), not -1.
13) equation (16): the last 2 terms are the same, one of them is probably a typo
14) equation (9) of the sup: N instead of n twice.
15) just after this equation "p(n,t|n0,t+delta_t)=p(N,t-delta_t|n+1,0)" does not make sense. I suppose "p(n,t|n0,delta_t)=p(n,t-delta_t|n0,0)" is what is meant.
16) equation (18) sup, I tried to redo, I think it's N^2, and not N.

We highly appreciate the careful reading of the manuscript and pointing out these typos (which did not propagate into the derivations and results of the manuscript). We have fixed these typos and have reviewed the rest of the manuscript to identify further errors, such as missing N that now appear in equations (60) and (61) of the Supplementary.

Smaller comments:

17) s values are huge! A 10 or 100-fold increase of fitness is not frequent, except in cases with a drug is

applied and the mutation confers drug resistance (and in which cases, realistically, there is often bactericide effects on top of the bacteriostatic effects)... In appendix, there is a smaller change of fitness, 3-fold, that is shown, but it is still really large. Maybe it would be good to also show the results (possibly in appendix) for more moderate (and more likely to be observed!) changes in fitness, let's say for instance 1.1.

We have added Figure S2 and a discussion of smaller fitness advantages to the Supplementary, which we reference in the main text.

18) line 338 "To provide a global measure of invasion success, we calculate the average invasion probability and the average MFPT to invasion, averaged over all possible initial locations of the invasion event. Surprisingly, this average invasion probability in the neutral spatial model is identical to the invasion probability of the neutral Moran model (s = 1) (i.e., it is equal to 1/N as shown in Fig 4C)." Actually I don't think it's surprising at all. In this model, the neutral invader can arise equally at each position in the channel. One of the N cells of the channels will be the ancestor of the whole population in the long term. Thus the invasion probability averaged on the initial position is 1/N.

We thank the reviewer for the intuitive explanation. We have added this explanation to the manuscript to elucidate the intuitive explanation of the mathematical results, at Line 353.

19) line 434: "the fixation time show a weak dependence on population size that is well approximated by an asymptotic logarithmic scaling in population size." : A logarithmic scaling in population size is plausible, but it's not fitted, and on such a small range (maximum 100 fold difference in N), it can be difficult to tease apart different possibilities.

We agree that with a 100-fold increase in N it is difficult to differentiate asymptotic scaling of the MFPT. The asymptotic scaling that we reference in line 434 arises from our heuristic approximation of the MFPT in the Results subsection titled "*Quantitative investigation of different dynamical regimes in large systems competing in a tug-of-war*", which is consistent with numerical results. We clarified this in the text.

20) page 17 in appendix, the paragraph commenting on fig 6C&E, the invasion probability for the Moran process being independent on N is not true for s=1.

We agree that this statement is incorrect and have removed it from the text.

***tiny comments**

21) fig 1C: the schematic is correct, but putting "death right" on the left side, and "death left" on the right side is kind of confusing on a first read.

We have changed the language such that the terms 'right/left cell removal' are used instead of 'death right/left'. We believe this change addresses the spirit of the reviewers' comments.

22) legend of fig 3B should give the reference of the equation for the dark dashed line.

We have added the reference to the equation for the dark dashed line in the caption of both Fig. 2B and 3B.

23) in the discussion in my opinion the initial summary of the findings should give a break down of the different models studied: case of a population with mutants on one side, WT on the other side; case of a mutant arising, either from an open end, or from anywhere with equal probability along the channel;

We have added a summary of the two cases (one boundary and two boundary problems) to the start of the Discussion, at Line 436. In this paragraph, we have also specified which characteristics of the dynamics are studied in each case, i.e. conditional or unconditional MFPT.

24) The first part of the appendix (pages 1 to 3) does not seem to relate directly to the rest of the manuscript.

We regret the confusion. Sections 1 and 2 of the Supplementary describe in detail the Master equation and the corresponding Fokker-Planck of our model, introduced in the Section Models and Methods of the main text. We hope this clarifies the reviewer's concern.

25) it would be good if references in the main text to the supplementary were more precise, towards a part of the supplementary, rather than to the whole thing.

We have implemented this suggestion to help readers better navigate the main text and supplementary.

26) fig 3A sup: the legend for the colors is put at a position where s is not visible.

The placement of the legend has been changed in light of this comment.

27) after 7.1, somewhere there is "thee" instead of "the"

We would like to thank the reviewer for their careful attention to the typing mistakes in the manuscript. We have done further read-throughs of the text to correct errors of this nature.

Response to Reviewer #2:

1) The abstract and introduction give the impression the authors are the first to theoretically study population dynamics incorporating pushing dynamics. But this is not the case. An early, important study in the context of cancer progression is Nowak, Michor, Iwasa, PNAS (2003), followed by Allen and Nowak, JTB (2012) https://www.sciencedirect.com/science/article/pii/S0022519311001809?casa_token=Sn7byKPwUxoAAAAA:NIY32ks8UAby_daQSqf2NPjiV__YmHNHhtVGv8TSVPgKPHcKljT92fP_Fs5OIud8ZlETneDtiA. It is more troubling that, in the introduction, the authors mention Koldaeva et al. (Ref. [66]) just as an experimental motivation for their study. In fact, Ref. [66] includes a systematic theoretical study of the neutral case - including exact expressions for the fixation probability and the logarithmic dependence of the fixation time on the system size. The authors should properly refer to the relevant literature and clarify what is new here.

We thank the reviewer for pointing out these studies, we have included a more comprehensive discussion of the relevant literature in our introduction and clarified what is new in our study.

Nowak and coworkers considered the standard mother machine geometry, where the cells can leave the channel from one end only. This geometry is completely different from ours and the results are significantly different: given that the founder cell at the closed end of the channel could not be displaced, it's lineage would assuredly fixate. We now cite them and have carefully emphasized this in the introduction.

Koldaeva et al consider a channel with two openings, akin to ours, but focus on a rather different quantity – average time to fixation of the lineage of one of the bacterial cells initially present in a one-dimensional lane. Within a number of approximations employed in the paper the dependence of this quantity on N emerges to be approximately logarithmic. However, we stress that this is an entirely different quantity compared to ours – fixation of one of the two species present initially regardless of progeny of how many individual cells remain present. The fact that it's also logarithmic is a appears to be coincidental.

We have clarified these issues in the Introduction (starting at Line 73) and throughout the text where appropriate.

2) I am quite familiar with the mathematical techniques used in this work and therefore could follow its logic. However, I feel that the authors made little effort to make the manuscript accessible to a broader audience. In particular, most of the results are presented in a rather technical fashion, without clearly explaining their biological significance. I feel that this is not the standard for PCB and I therefore recommend a substantial revision, having in mind readers that are less familiar with the math. One approach could be to separate the main messages of the paper from the mathematical derivations and analysis.

We thank the reviewer for these comments. We have revised our manuscript substantially to make it more accessible to a broader audience. In particular, we have moved the substantial fraction of the mathematical derivations and analysis to the Supplementary material, retaining in the main text only the minimum appropriate for the computational audience of PLoS Computational Biology. We have also revised all sections to provide a more concise summary of the mathematical models, methods, and their results in a non-technical language. Additionally, we have included more biological context and explanations of the significance of our findings throughout the text. We hope this satisfies the reviewer's concerns.

Other issues:

3) The Methods section begins with a lengthy review of the Moran model, which seems unnecessary since the model is so well established. This part can be drastically shortened. In contrast, the model with two boundaries used for Fig. 4 should be at least briefly defined in this section (although I agree with the choice of leaving the mathematical details for the SI).

We have significantly revised the Methods section, moving most of the discussion about the Moran model to the Supplementary Materials to provide a more concise and focused overview of the spatial model in the main text. Regarding the description of the two-boundary problem, we feel it is more appropriate to keep the model defined in the later section entitled "*Due to spatial exclusion, invasions are less likely to*

succeed on average and succeed on shorter timescales". Moving the description of the invasion two-boundary model to a later section allows the reader to differentiate the specifics of each model and helps separate the results. We also now more clearly distinguish between the two models in the Discussion.

4) Line 234, "grow only sub-pclynomially". The fixation time is actually logarithmic, as exactly proven in Ref. [66]. It is surprising that the logarithmic scaling is mentioned in the abstract, but in the results this logarithmic dependence is not really fleshed out.

The fourth and final subsection in our Results, titled *"Quantitative investigation of different dynamical regimes in large systems competing in a tug-of-war*", describes our derivation of the *log(N)* scaling. In light of this comment, we have added further clarifying remarks in that section.

We show that there is a logarithmic scaling in *N* of the fixation between two species at the equiprobable takeover abundance. In contrast, the logarithmic dependence of the fixation time on *N* derived in *Koldaeva et al.* is the mean time to fixation of a lineage in the channel – a very different fixation process (although denoted by the same name). As noted above, we have included a discussion of this distinction in the conclusion, emphasizing the need for further research to understand the relationship between the two.

5) Line 334-337 The fact that mutations in the center of the channel are much more likely to reach fixation is, again, exactly proven in Ref. [66], although only in the neutral case.

We have added a reference to this work in the appropriate section to inform the reader that our results support the results of this earlier work.
