# Supplementary material for: Spatial exclusion leads to “tug-of-war” ecological dynamics between competing species within microchannels
Source: PLoS Comput Biol. 2023 Dec 1;19(12):e1010868. doi: 10.1371/journal.pcbi.1010868 (PMC10718426; doi:10.1371/journal.pcbi.1010868)
Supplement: Attachment — Submitted filename: Response-to-Reviewers.pdf [file pcbi.1010868.s003.pdf]

Response to Editorial and Reviewer comments

We thank the Editor and both Reviewers for constructive comments (shown in blue). Our responses are in black.

Both reviewers agree that the manuscript is greatly improved from the previous version and are optimistic about it being able to be suitable for publication. They also both agree that the authors should do more to emphasize the continuity between the results of Koldaeva et al and those in the present manuscript. Reviewer 2 additionally points out that the responses to the two main points of their previous review are insufficient. First, small fitness differences are only treated briefly in the supplement, without the results being integrated into the main text. For instance, in the main text it states that (line 278) "higher fitness differences in the Moran model reduce the MFPT unconditioned on the success of a species", but this is not true for small fitness differences, as shown in the supplement. Second, several references to fixation times are still unclear.

Trying to synthesize all the reviews and my own impressions, I think that the manuscript needs to spend less time on trying to draw contrasts with other models and more on helping the reader gain an intuitive understanding of the model and its conclusions. The goal should be explain why every result is natural and makes sense. If the authors find any results to be surprising or counter-intuitive, they should try to see if there's an alternative framing that makes them intuitive.

We have revised the paper along the suggested lines, as detailed below and in the attached revised manuscript with highlighted revisions. In addition to the highlighted revisions, we re-arranged the order of some of the sections in the manuscript, in response to comment 12) of Reviewer 1.

A few specific suggestions to get started:

1. The key ingredient here is the biased pushing or whatever you want to call it. This creates strong spatial variation in reproductive value (Fig 4B, s=1). Cells in the middle get to reproduce for many generations before they have any chance at all of losing any indviduals, while cells on the edge are doomed. This manuscript deals with the consequences of this spatial variation for evolution. This needs to be explained as early as possible---if it's too complicated for the abstract, at least in the Introduction, and then with a simple intuitive description in the model section. And it should be included in Fig 1. (I would suggest replacing Fig 1 entirely with just an illustration of the collective pushing effect.) This should allow all the subsequent results to be explained fairly intuitively. (For instance, because the pushing induces an effective strong selection against cells at the edge, very strong selection is needed to allows cells to invade from the edge.)

We thank the Editor for the suggestion. We have amended Figure 1 accordingly, illustrating that the probability of the boundary to move towards the exit is biased because of the asymmetric division probabilities of the individual cells. Throughout the text, we now refer to this phenomenon as “skewed collective growth”, and emphasize that it underlies the main results of the paper in the Abstract, Summary, Introduction, Discussion and the appropriate places in the Results and figure captions.

2. When discussing MFPTs, there always needs to be a clear distinction between changes driven in by changes in dynamical time scales and those driven by changes in fixation probabilities. This is true even

in the second part where the manuscript is conditioning on invader fixation, because it is still averaging over different initial locations. For instance, in the neutral results shown in Fig 4D, I'm guessing that the dynamical time scales (i.e., the average fixation times for lineage originating at specific locations) are actually increasing with N or remaining constant, and the decrease in MFPT is because lineages in disfavored starting locations, whose fixation would be slower, have lower fixation probabilities as N increases. If this is true, I think that the presentation of MFPT here is going to tend to lead readers astray. Even if it's not true, the more important point is that the text should make it totally clear what's going under the hood. A similar comment applies for the effect of increasing invader fitness in the same panel.

We thank the Reviewer for this comment. We have clarified these differences throughout the text. We have also revised the figure in question based on the Editor's comments (please note that this is now Figure **5D** due to the re-structuring of the sections order in response to Comment 12 by Reviewer 1). In particular, we have changed how we calculate the average of the times of successful invasions over the initial location of the invader. Instead of each location being weighed equally, the times are now weighted by a more appropriate weight: the probability of invasion success at that location. These average times indeed increase with the system size and decrease with the fitness difference. In the neutral case, thus averaged successful invasion time scales approximately logarithmically with the system size similar to the lineage fixation time derived in Koldaeva et al, providing the connection to their work (please see also below response to Comments 2,5 by Reviewer 1 and Comment 3 by Reviewer 2). We revised the text accordingly to reflect these issues. We hope that the current version addresses the Editor's concerns.

Reviewer #1

We thank the reviewer for the careful reading.

1) in the answer to the comment 1 of the other reviewer, the authors state that "Koldaeva et al consider a channel with two openings, akin to ours, but focus on a rather different quantity – average time to fixation of the lineage of one of the bacterial cells initially present in a one-dimensional lane. Within a number of approximations employed in the paper the dependence of this quantity on N emerges to be approximately logarithmic. However, we stress that this is an entirely different quantity compared to ours – fixation of one of the two species present initially regardless of progeny of how many individual cells remain present. The fact that it's also logarithmic is a appears to be coincidental." I don't find the wording very clear. Koldaeva et al consider the diversity at a given time, and the time it takes so that one initial bacteria is the ancestor of all the bacteria in the system; they also calculate the invasion probability of one bacteria at a given position in the neutral case. The authors in this manuscript look at the probability for one bacterial type to take over, in the case with only 2 bacteria types. They also calculate invasion probabilities for one bacteria at a given position, but go beyond the neutral case.

Following the Reviewer's comments, we have appropriately revised the manuscript to more clearly explain the distinction between the two studies, in the Introduction, Model, Results and the Discussion as highlighted in the revised manuscript. In particular, we now provide a direct comparison of our invasion results (in the neutral case) with the results of Koldaeva et al in Figure 5D (see also response to Comment 2 below).

In brief, Koldaeva et al investigate the loss of complete lineage diversity in a neutral scenario in a channel where initially every cell belongs to a distinct (but otherwise identical) species. In a single lane, their results

describe the probability and average fixation time for the progeny of one of the initial bacteria taking over the entire lane.

By contrast, we focus on the case when only two distinct species are present in the channel, both in neutral and non-neutral regimes.

In our investigation of the invasion (2 boundary) problem, we calculate the first-passage time of one invader cell taking over the channel initially populated with another species, conditioned on a particular initial invasion location in the channel. As is now shown in Figure 5D, Koldaeva's average loss of diversity time is close to our time of a successful invasion averaged over all possible initial locations in the channel with appropriate weights.

Furthermore, one of the main results of our paper is the investigation of the 1 boundary problem, where two species that are initially present in two contiguous domains vie for dominance in the lane. In this case bacteria present in the final fixated population arise from different progenitors in contrast to the invasion problem.

We have revised the paper accordingly to clarify these issues.

2) I disagree that the logarithmic dependence is completely coincidental. Here they show that the time for fixation at the initial frequency which has the longest average time to fixation (the half/half for the neutral case) depends logarithmically on N. For the quantity looked at by Koldaeva, the time to fixation of a lineage from one of the N initially present bacteria, in most cases the bacteria which lineage fix comes from the middle of the channel, and thus the typical time it takes for this lineage to fix on one side will be the same ordre of magnitude than for it to fix on both sides. So, at least heuristically, it makes sense that both quantities have the same overall dependence on N.

We agree with the heuristic explanation suggested by the Reviewer and have added it to our revised text in the Discussion.

3) A point that I had not noticed in my first review, due to my insufficient reading of the Koldaeva reference. It concerns that (starting from line 203) "in open-ended micro-channels, it has been experimentally observed that cells are more likely to grow in the direction of the closer channel opening because fewer cells need to be pushed in that direction. The probability for a cell to grow towards one of the openings was observed to scale linearly with the number of individuals between the cell location and the other opening [57]" (57 is the Koldaeva reference). In this manuscript, pleft=(i-1)/(N-1), whereas in Koldaeva, pleft=im/(N-1)+(m(N+1)+N-1)/(2(N-1)), with m a parameter. If m=0, then there is no bias towards the end of the channels. If m=1, pleft=(N-i)/(N-1), which is, with some confusion on the meaning of left/right, the same than this manuscript. However, the fit in Koldaeva data gives m=0.6. The authors should state explicitly that they have chosen a limit of the theoretical analysis of Koldaeva, which is for somewhat larger impact of the numbers of individuals on each side than supported by the data.

We thank the Reviewer for pointing this out. We have extended the explanation to our description of the model in the Model and Methods section and the SI to better connect it to the previous work along the lines suggested by the Reviewer. We also added a discussion in the Discussion of the potential effects of the different choices of division rates in the model.

4) Paragraph lines 81-87. "Using heuristic analysis, the study showed that the diversity of the genetic lineage can quickly be lost in the channels where identical cells grow in lanes. However, a full mechanistic description of the dynamics of two species populations with fitness differences competing due to spatial exclusion in this one-dimensional space has not been elucidated." I don't think this is very fair to the Koldaeva study. They use some heuristics, but they also make some analytical calculations. And their model is also "fully mechanistic", though one very valid point is that they are not considering fitness differences, contrary to this manuscript.

We have revised the text to clarify this issue, which now reads "[*..] a recent study explored the lineage diversity of a microbial population in a channel with two open ends ~\cite{koldaeva2022population}, combining experiments and mathematical modeling. Focusing on the time of complete loss of lineage diversity in a system where at the initial configuration each cell belongs to a different species, the study revealed that lineage diversity can rapidly diminish within channels. However, the dynamics of fixation, extinction and invasion in a population of two species with fitness differences competing due to spatial exclusion in the one-dimensional space remain incompletely understood."*

We hope this adequately reflects the results of Koldaeva et al paper; please see also response to Comment 3 by Reviewer 2.

5) The authors could compare their solution (both probability and time to fixation) to the Koldaeva result for the invasion case of a neutral bacteria.

We thank the Reviewer for this suggestion and have now conducted a comparison between our solution and the Koldaeva et al result for the invasion case of neutral bacteria. Our analysis indicates that for the neutral scenario, Koldaeva's results are in good agreement with the exact solutions of our invasion model appropriately averaged over the initial location of the invader, which shows logarithmic scaling with N as is shown now in Figure 5D. We have further elaborated on the link between our findings and those of Koldaeva et al in the Introduction and the Discussion.

6) The study of cases with more reasonable fitness differences is a bit weak. They add one figure with less enormous fitness differences for the first question, but nothing for the invasion process (neutral, 10 fold, 100 fold more fit, which is enormous). This question of the fitness differences was stressed by the editor, and in my mind, the authors did really a minimal job on the subject.

We have replotted Figure 5 with the relative fitness (now denoted a w- please see response to Comment 7 by Reviewer 1 below) for the set of values 1, 1.5 and 10. For consistency we have also changed the values of relative fitness in Figure 2 (for the one boundary case) to 1, 1.5, 10, and 100. We have also expanded the discussion of the effects of the fitness difference in the text.

7) Besides, I am annoyed by the authors not answering properly to my comment 8. It was not a crucial comment, I was just commenting than in general, s is the notation not for the fitness itself, but for the difference in fitness: if one species has fitness "1", and the other one has a fitness of 1+s; whereas in this manuscript the reproduction rate is 1 for one species, and s for the other. The authors replied saying that they are using a convention used by other authors. They refer to 2 sources. In the PRE article they cite, actually when s is introduced, "In this spirit, we assume that the relative fitness of alleles X on island i is [WX]i = 1 + s ρi, and correspondingly [WY]i = 1 + s σi for allele Y on island i, where the strength of selection parameter, s, is small. ", which is actually exactly what I was suggesting, and *not* the

choice of the authors. They also cite a book. And in this book, I could find the notation s used in 3 examples. In one, it has nothing to do with fitness, s1 and s2 are some frequencies. In another one, s is indeed a fitness, as used in this manuscript by the authors, but I think that in that example s was chosen because it is the relative fitness of a superinfection. And there is a 3rd example where s is used, for a fitness 1 + s x, thus more in line with the usual notation of s as the relative increase in fitness. Notations are not that important, but it may be clearer for the readers to use more common conventions. I found the authors'answer unpleasant, because this gives the impression that either they did not care and put whatever references they had in mind without checking; or they knew their references were not particularly supporting to their choice of notation and thought I would not check.

To remove any ambiguity, we have renamed the relative fitness difference (previously denoted by "s" in our paper) to "w", in particular to avoid any confusion with the common usage of "s" denoting the selection coefficient ($w = 1 + s$). We hope that this change addresses this issue.

*** Though it is really quite improved compared to previous version, I think there are still some places where some aspects are unclear.**

8) In the abstract: "In non-neutral cases, (...) and the maximal system fixation times increase as the relative fitness differences between species increase." is confusing for 2 reasons: it does not say that this fixation time is not conditioned on the success of one particular species, and it does not say maximal relative to what (it is the maximal unconditional fixation time, relative to the location of the initial boundary between species). I'm not sure the result is so fundamental to be stated in the abstract, given the space it takes to state it properly.

We have revised the Abstract to provide a more precise description of our findings.

9) I reiterate that I think that stating that the results are in 1-dimension is important enough to be mentioned in the authors' summary, and not just the abstract. The sentence "In this study, we theoretically and computationally study the competitive dynamics of two bacterial populations competing for space in confined environments" could be transformed to something like "In this study, we theoretically and computationally study the competitive dynamics of two bacterial populations competing for space in a dimensional confined environment".

We revised the Authors’ Summary accordingly to explicitly state that our results are for one dimensional lane.

10) paragraph lines 525-538. The discussion there is framed as if there were some trade-off between fitness (replication rate) at a given time and "strain on metabolism" and "resource costs" (that would imply slower replication at a later time?). I am unsure of what relevant examples would be for bacteria.

Moreover, it is framed around the increase with fitness of the time to fixation (of either species) at the equiprobable takeover initial abundance; however, the time to fixation conditioned on success, at a given initial abundance, is always smaller for a higher fitness. I don't understand the message that this paragraph is supposed to convey.

In their answer to my point they numbered 6, the authors state that "What’s more, the unconditional time to fixate at the equiprobable takeover abundance increases with fitness difference. At this abundance, the fitter species has the same probability to successfully takeover as the less fit species,

yet the competition now lasts longer than at lower fitness differences. The populations must make a decision on the strategy to undertake based on the trade-off between higher success in the chamber and shorter competition times." I really disagree on this. Let's suppose that the population is able to make a decision (which can be doubtful for bacteria). At a given initial relative abundance of one species, then an increase in fitness does increase the chance of success and does reduce the time to fixation conditioned on success. It is only looking at the initial abundance for equiprobable takeover, that shifts with fitness, that the conclusion is reversed.

To avoid confusion, following the Reviewer's comments we have completely rewritten this paragraph, avoiding the language of "decision-making".

11) Around line 304-311 of the document with highlighted changes: "Counter-intuitively, in the spatial exclusion model, although a greater fitness is generally associated with a higher likelihood of population fixation, increased fitness does not lead to faster fixations.". I think it would be clearer to use all the results and say something like: "In the spatial exclusion model, though a greater fitness always leads to faster fixation conditioned on the species success (see fig 4B of the supplementary material), this is not always the case when considering the unconditional fixation probability."

We have changed the wording in the section to include this distinction between the conditioned and unconditioned MFPT.

12) section "Quantitative investigation of different dynamical regimes in large systems competing in a tug-of-war": is it back to the case where one side is one species, the other the other species? Or are we still in the invasion problem as in the section just before? If it's the one boundary system, it would be much clearer for the reader to say explicitly at the beginning of this section that it is now back to the first set-up. If it's the invasion set-up like previous section, then many things don't make sense.

The model in this section is indeed the one boundary case. To make this clearer to the readers, we have moved this section up, before the introduction of the two-dimensional invasion model.

13) Overall in this manuscript, the level of detail of the calculations is ok, but I found it very hard to follow the calculations of the supplementary part 7.

We thank the Reviewer for their feedback. We have rewritten the section to better guide the readers in our heuristic approximation calculation.

* Other comments (random order).

14) their reply to my comment 15: they did make a change, but it is still not ok. It is not true that the transformation is (neither should be!) p(n, t|n0 , t + ?t) = p(n, t ? ?t|n0 , 0), it's p(n, t|n0 , ?t) = p(n, t ? ?t|n0 , 0).

We corrected this error as well as a few other typographic errors found in other sections of the manuscript.

15) Figure 4 of the sup: how many simulations for calculating the conditional fixation time? Any indication of the standard deviation between first passage time? (I ask because when the low fitness species starts at a low frequency, its probability of fixation is very small).

As with the rest of the paper, these results are numerical solutions found not by stochastic simulations but by the numerical solution of the Fokker-Planck equation, which was verified to provide a very close approximation to the direct matrix inversion of the master equation (please see Section 4 of the SI). We have now specified this in the caption of the figure and more clearly state it in the Methods and the Results. We thank the Reviewer for pointing out an interesting future direction of inquiry, however we feel that calculation of higher moments of the fixation times is outside of the scope of the present manuscript.

16) Frankly I don't think that part 5 of the appendix is interesting: averaging on the initial frequencies, with uniform probability. Why this choice? This gives some aggregate number, but I don't think it has any physical meaning.

One of the challenges faced in experimental settings is the inherent variability in initial conditions. In many real-world scenarios, it's often impractical or even impossible to precisely control or fix the initial frequency of a system. This variability arises from a range of factors such as environmental fluctuations, or other stochastic effects. The averaging in Section 5 allows us to quantify the system's behavior across a spectrum of initial conditions. By obtaining an aggregate result through this averaging process, we can provide benchmark results that can be compared with experimental outcomes (such as in microfluidic realizations of these systems). We have added an explanation of this motivation in Section 5 in the SI. We feel that this calculation completes the paper as a part of the supplementary results.

17) line 307: "location dependent" -> it's not the location, but the initial frequency f. That frequency translates in a location for the interface, as here it is the case when each species is on one side, but location is more relevant for the invasion case.

We have clarified that the location of the boundary between the two species is synonymous with the initial frequency f that is referred to on line 307.

18) Equation just after line 324 should be with f instead of x

The equation appearing in the highlighted file of the previous submission is in red, indicating that it had been deleted in the main file and does not appear in the revised manuscript.

19) line 422 "a regime relevant for many experimental systems that are often comprised of large numbers of individuals" Could you be more explicit about such systems? Microfluidic channels are usually not very long, mostly for nutrients to diffuse enough.

We've added references to the systems that may exhibit similar geometries and include large number of bacteria: crevices in biological systems (e.g. gastrointestinal crypts) and narrow channels in which bacteria may move and grow (e.g. Antarctic icesheets). We also note that numerically the approximation is very good already in the range $N \simeq 100$

20) Figure 5b. The legend directly on the figure does not match the change in variable names.

We have made the appropriate change from x_{max} to f_{eq} and fixed similar typos throughout the text.

21) The discussion paragraph between lines 572 to 580. First line 573 it mentions invasion, then line 576 it mention than in this manuscript there is an "exact solution" for the MFPT (that decreases with N). By

exact solution, it is meant the numerical solution? Because the analytical solution showing the logarithmic dependence on N is an approximation, according to equation 12.

The exact solution is indeed an exact numerical solution involving matrix inversion of the master equation, as discussed in the Supplementary. We've now clarified this in the text.

<u>**Reviewer #2:**</u> **The authors successfully addressed most of my comments and the manuscript has greatly improved.**

We thank the Reviewer for the positive assessment.

1) I have some remaining concerns about the logarithmic scaling of the fixation time. In particular, I have not understood the argument by the authors, according to which the mean fixation time as defined in Koldaeva et al is a different quantity than that studied in this manuscript. I do understand that the initial condition is different: an infinitely diverse initial condition (with each individual belonging to a different strains) in Koldaeva et al. versus two strains involved in a "tug-of-war" here. But this is not really clear from the manuscript, and it would be better to express it in simpler terms.

We regret that this was unclear. Koldaeva et al indeed measure/calculate the fixation time for the progeny of a bacterium to take over the channel and provide a calculation of this time based on their theoretical model (please see response to Comment 3 below). This time closely corresponds to our invasion time averaged over the initial conditions weighted by the probability of fixation from that initial condition. This is now shown in Figure 5D. This scenario is different from the case of two strains involved in the tug of war shown in Figure 2, 3 and 4 as is now appropriately explained in the accompanying text.

Following the suggestion of both Reviewers, we have added a paragraph to the Discussion that explains the similarities and distinctions between the different scenarios in our and Koldaeva et al.'s work.

2) I also do not think that it is surprising that the log(N) scaling is common to both cases. The common factor is the accelerating dynamics of interfaces far from the center of the channel. This is the case regardless of the initial condition.

We agree with the Reviewer that the accelerating dynamics far from the center of the lane is a commonality between the two models that leads to similar behaviour (see also response to Comment 2 of Reviewer 1)

With insights from both Reviewers, we have added the appropriate discussion around Figure 5D and in the Discussion.

3) Please also note that Koldaeva et al. provides an exact expression for the mean fixation time in the one-lane case. Therefore, the expressions "Using heuristic analysis" at line 83 and "Using simplifying assumptions" at lines 527-528 are not correct.

We thank the Reviewer for this comment and regret the confusion. To the best of our understanding the Koldaeva et al. derivation of the logarithmic dependence of the lineage diversity loss time on N relies on auxiliary ansatzes, such as the statement the the diversity loss times to go from A to $A-1$ lineages are exponentially distributed (e.g. Koldaeva et al, SI section *Mathematical derivations: First regime of diversity loss*). While we cannot exclude the possibility that this might be correct specifically for the initial conditions of Koldaeva et al, we find that this cannot be correct in general. For instance, it is clearly

violated for transition from 2 to 1 species for almost any initial condition, as shown in the attached Figure (which is not included in the manuscript) – simply because there is typically a delay for the boundary to diffuse towards the absorbing exits. However, we feel that further discussion of these technical issues is out of the scope of the current manuscript.

We now show the comparison between Koldaeva et al. and our calculations in Figure 5D. We have also revised the introduction to read *“[...] a recent study explored the lineage diversity of a microbial population in a channel with two open ends ~\cite{koldaeva2022population}, combining experiments and mathematical modeling. Focusing on the time of complete loss of lineage diversity in a system where at the initial configuration each cell belongs to a different species, the study revealed that lineage diversity can rapidly diminish within channels. However, the dynamics of fixation, extinction and invasion in a population of two species with fitness differences competing due to spatial exclusion in the one-dimensional space remain incompletely understood.”*

We hope that the revised manuscript fairly reflects the results of Koldaeva et al and their relation to our work.

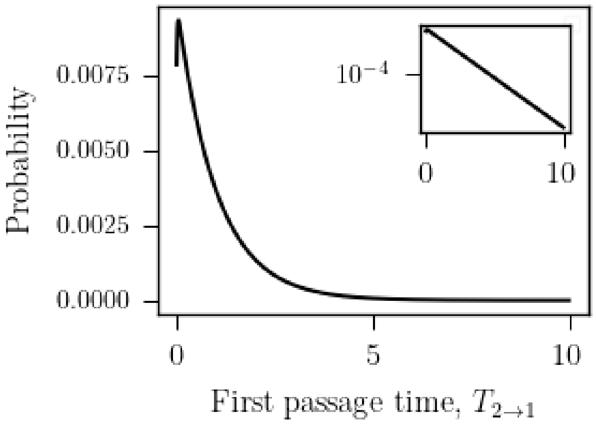

Probability distribution of fixation times to go from 2 species to 1 species in a 1 boundary model, showing the non-exponential regime at short times.
